# The Role of Copper in the Hydrogenation of Furfural and Levulinic Acid

**DOI:** 10.3390/ijms24032443

**Published:** 2023-01-26

**Authors:** Cristina García-Sancho, Josefa María Mérida-Robles, Juan Antonio Cecilia-Buenestado, Ramón Moreno-Tost, Pedro Jesús Maireles-Torres

**Affiliations:** Departamento de Química Inorgánica, Cristalografía y Mineralogía (Unidad Asociada al ICP-CSIC), Facultad de Ciencias, Campus de Teatinos, Universidad de Málaga, 29071 Málaga, Spain

**Keywords:** furfural, levulinic acid, hydrogenation, hydrogenolysis, MPV reaction, copper catalysts, furfuryl alcohol, 2-MF, γ-valerolactone, cyclopentanone, 2-methyltetrahydrofuran

## Abstract

Currently, there is a great interest in the development of sustainable and green technologies for production of biofuels and chemicals. In this sense, much attention is being paid to lignocellulosic biomass as feedstock, as alternative to fossil-based resources, inasmuch as its fractions can be transformed into value-added chemicals. Two important platform molecules derived from lignocellulosic sugars are furfural and levulinic acid, which can be transformed into a large spectrum of chemicals, by hydrogenation, oxidation, or condensation, with applications as solvents, agrochemicals, fragrances, pharmaceuticals, among others. However, in many cases, noble metal-based catalysts, scarce and expensive, are used. Therefore, an important effort is performed to search the most abundant, readily available, and cheap transition-metal-based catalysts. Among these, copper-based catalysts have been proposed, and the present review deals with the hydrogenation of furfural and levulinic acid, with Cu-based catalysts, into several relevant chemicals: furfuryl alcohol, 2-methylfuran, and cyclopentanone from FUR, and γ-valerolactone and 2-methyltetrahydrofuran from LA. Special emphasis has been placed on catalytic processes used (gas- and liquid-phase, catalytic transfer hydrogenation), under heterogeneous catalysis. Moreover, the effect of addition of other metal to Cu-based catalysts has been considered, as well as the issue related to catalyst stability in reusing studies.

## 1. Introduction

**Scheme 1:**
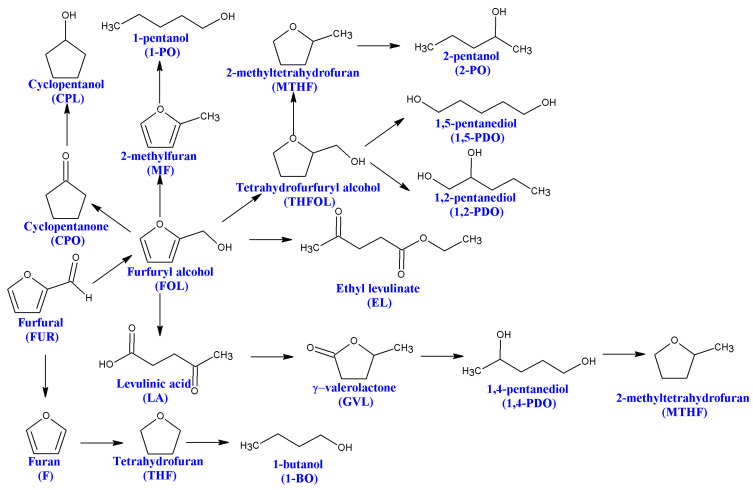
Catalytic routes of hydrogenation of furfural.

In the last decades, much attention has been paid to the development of suitable technologies for the conversion of nonedible lignocellulosic biomass into biofuels and chemicals, as a sustainable and renewable alternative to fossil resources. In this context, nowadays, furfural (FUR) is considered by the US Department of Energy (DOE) as one of the top 12 value-added products derived from lignocellulosic biomass, with a world production of about 300,000 Tm/year [1,2]. FUR is obtained from the hydrolysis of hemicellulose and subsequent dehydration of the resulting monomeric xylose, and the interest in this platform molecule, the second-most demanded after bioethanol from biomass, lies in its high reactivity [3]. The presence of an aldehyde group and a furan ring can lead to the formation of a wide range of derivatives by hydrogenation, oxidation, alkylation, opening ring, and condensation, among others. In the case of FUR hydrogenation, catalytic processes have been reported in both gas- and liquid-phase using transition-metal-based catalysts, as well as by catalytic transfer hydrogenation with acid/base catalysts [3,4,5]. In all cases, the selection of the active phase plays an important role in the catalytic activity, mainly determining the selectivity pattern. Thus, metal catalysts with higher hydrogenating capacity, such as Ni, Pd, Pt, Ru, or Rh, are highly active in FUR hydrogenation, although frequently give rise to uncontrolled reactions, where yields towards a target valuable product can be relatively low. However, catalysts with lower hydrogenating capacity, such as Cu-based ones, are less active, in such a way that the number of products is more limited due to the inability to break C–C bonds in the FUR structure. Under these later conditions, the main products are generally 2-methylfuran (MF) and furfuryl alcohol (FOL), which exhibit high potential due to their industrial applications (Scheme 1).

The nature of copper species (oxidation state, nanoparticle size, interaction with other metal in bimetallic CuM-based catalysts, existence of surface oxygen vacancies, morphology, and copper–support interaction depending on the acid–base properties of support, among others) can exert a strong influence on catalytic activity in the hydrogenation of furfural and levulinic acid, determining both conversion and selectivity pattern, in addition to its effect on the stability of the active phase. The advantages of copper compared to noble metals (Pd, Pt, Ru, Au) often used in hydrogenation processes are easy accessibility, worldwide distribution, and competitive price, which make Cu-based catalysts very promising hydrogenation catalysts. However, in some cases, there is a lack of consensus about the required oxidation states (Cu^0^, Cu^+^) to achieve a better catalytic performance, although both species could be involved, in such a way that Cu^+^ species, as Lewis acid sites, interact with the oxygen of carbonyl group of FUR, whereas metal copper ones dissociate hydrogen molecules. Moreover, the process assisted by ultrasound or microwave irradiation instead of conventional heating can improve conversion, whereas weak acid sites favor the adsorption of furfuryl alcohol and its subsequent hydrogenolysis to MF. In the case of reducible supports, such as CeO_2_, oxygen vacancies associated with the different oxidation states of Ce can modify the electronic density of copper nanoparticles, leading to different interactions with furfural and levulinic acid molecules, and, consequently, to different hydrogenation products. Therefore, there are different strategies enabling to attain a suitable catalytic performance, inasmuch as the nature of surface active species can be tuned.

FOL is used in the manufacture of foundry resins due to its excellent thermal stability, chemical, and mechanical properties, for the manufacture of plastics and nonpetroleum chemicals, as well as pharmaceutical and agrochemical products. MF, also known as sylvan, is a flammable, water-insoluble liquid with a typical odor, useful as solvent and as feedstock to produce antimalarial drugs (chloroquine), crysanthemate pesticides, perfume intermediates, 2-methyltetrahydrofuran (MTHF), and functionally substituted aliphatic compounds, among others. MF itself presents interesting properties as alternative fuel in the gasoline range, with an octane number of 74 [6], and a thermal efficiency higher than gasoline and 2,5-DMF, due to its fast burning rate and notable better knock-suppression ability [7]. Another reduction product derived from furfural is cyclopentanone (CPO), which is a valuable chemical intermediate for the production of medicines, herbicides, pesticides, fragrances, fuel precursors, and high-density fuels after condensation–hydrogenation processes [8]. It is a colorless liquid with a slightly peppermint odor, which is soluble in water and miscible with common organic solvents [9]. However, it is industrially obtained by the decarboxylative cyclization of adipic acid or by the direct oxidation of cyclopentene [10].

On the other hand, levulinic acid (LA), which can be obtained from cellulose, is the starting point for the synthesis of a portfolio of high-value-added chemicals, such as γ-valerolactone (GVL) [11,12], 1,4-pentanediol (1,4-PDO) [13], MTHF [14], and ethyl levulinate (EL) [15], among others (Scheme 1). LA prices, which currently range between USD 5 and 8/kg, should be reduced by about USD 1/kg to penetrate downstream applications [16]. 

γ-valerolactone (GVL) is considered to be a versatile building block for the production of valuable chemicals and high-grade fuels derived from renewable feedstocks [17,18,19]. It can also be used as precursor to produce fragrances, food additives, green solvents, polymers, and value-added chemicals, such as 2-methyltetrahydrofuran, methyl 4-methoxypentanoate, adipic acid, and aromatic hydrocarbons [20,21]. Moreover, it has been proposed as a fuel additive of gasoline and diesel, because GVL improves combustion properties due to its lower vapor pressure and reduces CO and smoke emissions in automobile exhaust [22], or it can even be directly utilized as liquid fuel due to its similar heating value and higher energy density than ethanol [23]. Considering this great potential, numerous synthetic routes have been evaluated to obtain GVL in recent years, mainly from hydrogenation of levulinic acid (LA), since this latter can be directly obtained from lignocellulosic biomass by acid hydrolysis [19,24,25]. Recently, the development of efficient hydrogenation catalysts to convert LA to GVL has received considerable attention. Most studies have been focused on LA hydrogenation by using noble metal catalysts, mainly Ru-based ones [26,27]. However, due to their high cost, other inexpensive metal-based catalysts have been proposed in the literature. Considering that Cu-based metal catalysts for hydrogenation reactions are well documented, currently there is a trend to use them for LA hydrogenation to GVL [28,29]. As mentioned, in addition to a lower cost of copper compared to Ru or other noble metals, copper-based catalysts have been shown to be active in the catalytic transfer hydrogenation (CTH) reaction of LA to GVL, avoiding the use of high H_2_ pressures as reductant, as will be shown below.

Another of the key downstream potential applications for LA is the manufacture of MTHF. It is estimated that LA manufacturers could face a potential demand of over 20,000 kilotons from MTHF applications by 2020 [30]. MTHF is a versatile cyclic ether, an aprotic and hydrophobic organic solvent, with a low solubility in water; moreover, it is inversely soluble in water, i.e., the solubility decreases with the increasing temperature [2], with a high boiling point (80.2 °C), excellent stability, and it can be degraded by sunlight and air [31]. MTHF possesses no mutagenicity nor genotoxicity characteristics and the human permitted daily exposure limit is 6.2 mg/day [32]. Due to those favorable characteristics, it is used as a green substitute for THF [33].

MTFH is also considered a nonpetroleum liquid fuel with special characteristics, such as high energy density, lower flammability, hydrophobic nature, low toxicity, and high specific gravity, making it an adequate fuel [34]. Thus, MTHF can be blended with gasoline up to 70%, and it was approved in the USA as an oxygenated gasoline additive [35]. Despite its lower octane number (87), MTHF is a more suitable biofuel than ethanol, and it can greatly reduce the vapor pressure of ethanol when co-blended in gasoline. This has resulted in the development of “*P-Series*” fuels, where MTHF acts as co-solvent. Briefly, the *P-Series* fuels are mixture of ethanol, pentanes (hydrocarbons from natural gas with more than four carbon atoms), and MTHF. These fuel formulations contain from 64 to 70% renewable chemicals, provided by both ethanol and MTHF, which can be derived from renewable sources.

This review will focus on the catalytic activity of copper-based catalysts and the role of copper species in the hydrogenation of FUR and LA by conventional reduction with hydrogen and the CTH reaction. Other excellent reviews are devoted to other topics related to the reduction of bioaromatic compounds such as FUR or 5-hydroxymethylfurfural [36,37,38,39,40]. The authors are concerned that biocatalytic systems and electrocatalytic processes are being developed for these reactions, but they are out of the scope of this review. Readers can find recent reviews of these topics in the bibliography [41,42,43,44,45]. 

## 2. Hydrogenation of Furfural

### 2.1. Hydrogenation of Furfural to Furfuryl Alcohol

The first study in the gas-phase FUR hydrogenation was reported in 1929 using Cu/asbestos as catalyst [46]; then, Du Pont de Nemours Co. (Wilmington, DE, USA) patented the copper chromite as catalyst precursor for this reaction [47]. In the next decades, Quaker Oats Co. (Chicago, IL, USA) developed a Cu/Na_2_O_x_SiO_2_ catalyst, attaining a maximum FOL yield of 99% at 177 °C [48]. Currently, copper chromite (CuCr_2_O_4_·CuO) is the catalyst precursor used industrially in the gas-phase FUR hydrogenation [3]. The nature of active Cu sites has been at the center of debate in the last years, mainly centered on the role of Cu^0^ and Cu^+^ species in the hydrogenation process [49]. In this sense, using copper chromite, it has been proposed that both Cu^0^ and Cu^+^ species play an important role, where Cu^0^ species activate H_2_ and the Cu^+^ centers interact with the carbonyl group of FUR [50]. A second-order process was postulated to explain FUR hydrogenation, following a Langmuir–Hinshelwood model based on competitive adsorption between FUR and H_2_ by the same available active, or two specific sites, i.e., one for H_2_ and another one for FUR. In addition, the adsorption on Cu sites did not appear to be the limiting step, but rather the hydrogenation of FUR to FOL. More recently, Liu et al., also evaluated the role of both copper species using advanced experimental techniques [51]. Thus, XAFS studies demonstrated that Cu^0^ seemed to be the unique Cu species in the fresh catalyst, remaining as such throughout the entire reaction process. In the absence of other oxidation states, Cu^0^ must be the only active species in the gas-phase FUR hydrogenation. Moreover, they also observed a strong deactivation because of the strong interaction between FUR and Cu^0^ sites, which limits the desorption of products and the availability of active sites. In a later study of this research group, they deposited a thin Al_2_O_3_ layer using atomic layer deposition (ALD) to favor the regeneration of the catalyst upon calcination, without observing leaching or sintering of the active phase [52]. At short reaction times, the ALD-based catalysts showed a catalytic activity much lower than copper chromite, although these catalysts were less susceptible to deactivation because coke formation is minimized. The incorporation of ALD also affected the reducibility of copper chromite, in such a way that the coexistence of Cu^0^ and Cu^+^ species was observed, the activity being correlated with the Cu^+^ sites, so that these authors suggested that both Cu^0^ and Cu^+^ sites are necessary in the gas-phase FUR hydrogenation. In this sense, Cu^+^ species can act as electrophilic (Lewis acid sites) to polarize the C=O bond via an electron lone pair of oxygen, providing an additional interaction between the FUR molecule and Cu^+^ sites [53,54].

In the last decade, the environmental concerns related to the presence of chromium in copper chromite have led to the development of Cr-free catalysts. One of the most studied catalytic systems is based on small copper nanoparticles dispersed on a commercial silica, or mesoporous silica. From a mechanistic viewpoint, the first study of Cu/SiO_2_ catalysts in gas-phase FUR hydrogenation was carried out by Resasco’s research group [55,56]. They stablished that gas-phase FUR hydrogenation follows a Langmuir–Hinshelwood kinetic model, assuming the following premises: (a) molecular adsorption of FUR, FOL, and MF, (b) dissociative adsorption of hydrogen, (c) all adsorption sites are equivalent and independent of coverage, and (d) surface reaction is the rate-determining step.

The application of the Arrhenius equation revealed that FUR hydrogenation requires an adsorption heat of 11.8 kcal/mol, while hydrogenolysis of FOL is 12.4 kcal/mol [55], which are in the same range to those obtained for copper chromite [50]. They also established an adsorption heat for H_2_ and FUR of 13.9 and 12.3 kcal/mol, respectively. This latter value is higher than that obtained for FOL (6.9 kcal/mol), which was ascribed to the interaction of the carbonyl group of FUR with Cu sites stronger than that of the hydroxyl group of FOL. These values are in agreement with those previously reported in the literature [57]. In the case of MF, the adsorption heat is much lower, since the methyl group of MF barely interacts with Cu sites. This step (FOL → MF) involves the formation of H_2_O as byproduct, whose adsorption heat is 12.4 kcal/mol on Cu/SiO_2_ [55], so there is a competitive adsorption between FUR and H_2_O molecules by the Cu sites. Based on the adsorption heats, Shi et al., pointed out that, as FUR and H_2_ display similar values, the FUR → FOL reaction obeyed a Langmuir–Hinshelwood mechanism due to H species, and FUR molecules can be co-adsorbed on the surface of Cu/SiO_2_ catalysts [58]. However, the lower adsorption heat of FOL suggests that the FOL → MF should proceed via an Eley–Rideal mechanism.

Liu et al. [59], using DFT and microkinetics studies, were able to demonstrate which facets were more prone to adsorb FUR and FOL and activate H_2_ for different transition metal surfaces, concluding that the stepped (211) surfaces were more active for Cu, Ag, and Au, and terrace (111) surfaces were more active for Pt, Pd, Ir, NI, Co, Rh, and Ru.

**Figure 1:**
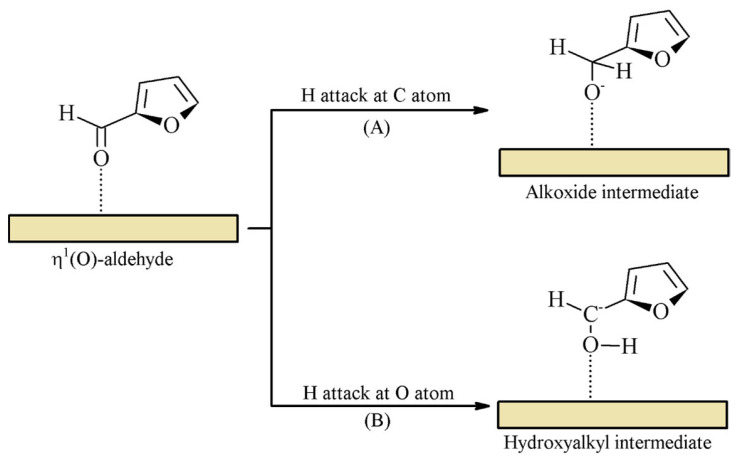
Orientation of FUR molecule on metal surfaces via formation of η^1^(O)-aldehyde species.

On the other hand, the combination of theoretical and DFT calculations clearly show that the strongest interaction of FUR with the Cu surface is via the lone pair of oxygen to produce η^1^(O)-aldehyde species, through both Cu(111) and (110) planes (Figure 1) [55,56]. However, the furan ring is not strongly bound to the surface, but, rather, it exerts a repulsion that increases with the surface electronic density of Cu atoms, probably due to the overlap of the 3*d* band of the surface Cu sites and the anti-bonding orbital of the aromatic furan ring.

From the DFT data, Sitthisa et al., predicted that, after the interaction of Cu sites with FUR via η^1^(O), the reaction can proceed through two possible pathways, where H, coming from H_2_ dissociation, can attack (i) the carbon of the carbonyl group, to generate an alkoxide intermediate, and (ii) the oxygen of the carbonyl group, leading to hydroxyalkyl species [55]. Nevertheless, the formation of the hydroxyalkyl intermediate is favored due to its stabilization with the furan ring. Other authors have confirmed that adsorbed aldehyde/ketone groups on Cu (111) are hydrogenated irreversibly by surface hydrogen to form an alkoxy intermediate [60].

More recently, Shi et al., established a detailed study related to the full potential energy surface for the production of MF from FUR on Cu (111) surfaces [58]. These authors pointed out that a homolytic dissociation of H_2_ takes place. Then, an H atom reacts with the FUR molecule, via η^1^(O)-aldehyde conformation, to form an alkoxyl group, this step being kinetically and thermodynamically favorable. Later, another H atom gives rise to the formation of FOL; however, this step is the limiting step in the gas-phase FUR hydrogenation using Cu/SiO_2_. In the reaction FUR → FOL, the high surface hydrogen coverage in FUR hydrogenation appears to prevent FUR dissociation. In the next step, the FOL molecule suffers a dissociation into alkyl and OH groups, subsequently giving rise to a molecule of H_2_O as a byproduct. In order to improve the MF yield, it is also necessary to tune the catalytic systems to modify the surface hydrogen coverage to enable the co-adsorption of FOL and hydrogen, as well as to remove the formed H_2_O from the surface. If there is H_2_O in the feed, the alkoxy intermediate is converted into FOL (via proton transfer) from either OH* or H_2_O* [60].

On the other hand, high MF yields have been reported by several authors using Cu/SiO_2_ catalysts [61,62]. It has been demonstrated that the presence of acid sites favors the hydrogenolysis of FOL to MF. In this sense, both pure CuO and SiO_2_ hardly display acidity. After its reduction, Cu/SiO_2_ catalysts generate a high amount of weak acid sites, which can be associated with the formation of Cu^+^ species due to a strong Cu–support interaction, as a consequence of the high electron affinity of silicon. This fact leads to an electron transfer from the Cu^0^ to silica, increasing the electropositive (or oxophilic) nature of copper, and also improving the oxophilic nature of the whole catalytic system, which favors the hydrogenolysis of the saturated C–O bond [61]. The reaction temperature is another key parameter that determines the selectivity pattern; thus, computational studies have demonstrated that the formation of FOL is favored at lower temperatures, while MF is at higher ones [58]. These theoretical data are in agreement with those obtained experimentally with copper chromite [51].

**Figure 2:**
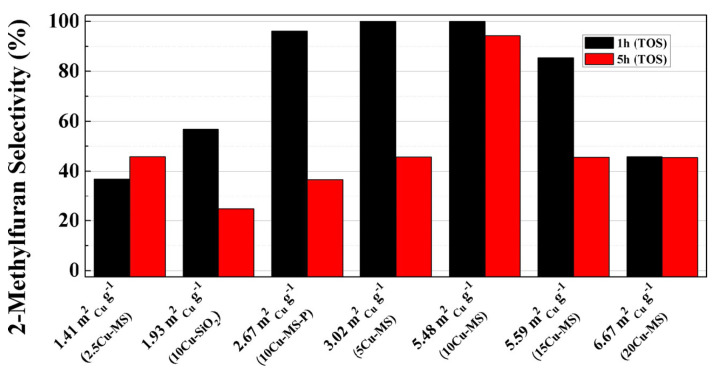
Influence of the surface area on the MF selectivity. Reprinted with permission from [62]. Copyright 2017 John Wiley and Sons.

Together with the presence of acid centers, another important parameter to favor the formation of MF is the existence of small Cu nanoparticles, which must be well dispersed on the support. Thus, Jiménez-Gómez et al., found an optimum metallic surface, about 5 m^2^_Cu_ g^−1^, to maximize the formation of MF *(*Figure 2) on copper supported on a mesoporous silica catalyst [62]. 

At shorter reaction times, this catalytic system is more active in FUR hydrogenolysis (MF formation), but carbonaceous deposits formed along the reaction cause the occlusion of channels, leading to diffusional problems and a strong deactivation of active sites involved in the hydrogenolysis process of FOL to MF, in such a way that a decrease in the MF selectivity is concomitantly accompanied by an increase in FOL yield. Another interesting alternative to obtain Cu nanoparticles dispersed on SiO_2_ was proposed by Liu et al., with the synthesis of ultrafine multiple core–shell-structured leaf-shaped Cu–SiO_2_ catalysts with small particle size and high copper loading [63].

In spite of most theoretical and mechanistic studies performed on commercial SiO_2_, other metal oxides have also been used to disperse Cu particles, obtaining an excellent catalytic behavior in gas-phase hydrogenation of furfural. Computational models have revealed that the binding energy for the attachment of Cu nanoparticles on the surface of MgO is larger (24.66 kcal/mol) than that shown for SiO_2_ (8.16 kcal/mol), so the copper nanoparticles are adsorbed more strongly on MgO [64]. In earlier studies, Nagaraja et al., pointed out that the high catalytic activity could be ascribed to the existence of oxygen vacancies, probably in the boundary grains, generating defective sites in the Cu–MgO interfacial region, increasing its catalytic activity [65]. Similarly, Ghashghaee et al., reported that the coordination number of the of oxygen atoms at the surface of the MgO crystals is lower than that of the interior O species, favoring a higher adsorption of extra-framework atoms [64]. In addition, these authors stated that the addition of H species to the C atom of the carbonyl group is more favored than its interaction with the adjacent O atom. An analysis of partial charges established that Cu species acquire a positive charge upon interaction with the carbonyl group, owing to a π-back donation from Cu to the C=O bond. In this sense, the presence Cu^δ+^ can provide Lewis acid sites, which can favor the hydrogenolysis reaction of FOL to MF [61]. Nagaraja et al., also found that the formation of small Cu species highly dispersed on MgO, prepared using a precipitating method, calcination and subsequent reduction, increased the amount of available Cu metal sites, while the presence of small MgO crystallites also generates a higher proportion of O vacancies [66]. More recently, Sadjadi et al., evaluated the influence of the precipitating agent on the dispersion of Cu species, and, consequently, on the catalytic performance [67]. They observed that the catalyst prepared by using NaOH as precipitating agent attained the highest conversion values, and the use of Na_2_CO_3_ and K_2_CO_3_ gave rise to catalysts with slightly lower catalytic activity, while the least efficient catalyst was that prepared using ammonium carbonate. In the same way, these authors compared between nitrate, sulfate, and acetate salts of Cu(II) to find out the role of the anion, proving that all catalysts were acceptably selective and active, although the highest performance was reached with the nitrate precursor [67]. Other authors have evaluated the promoter effect of Co^2+^, Ca^2+^ of Cr^3+^ species in Cu/MgO catalysts, pointing that Ca^2+^ slightly improved both the FUR conversion and FOL selectivity, in comparison to the unpromoted Cu/MgO, probably due to the generation of basic sites. However, the addition of Co^2+^ or Cr^3+^ hardly modified the catalytic activity, although the FOL selectivity slightly increased [68,69].

Several authors found that Cu species highly dispersed on ZnO also displays an excellent catalytic behavior, being mainly selective to FOL and resistant to deactivation processes [70]. In this sense, a partial reduction of Zn^2+^ species into Zn^δ+^ on the surface of Cu/ZnO catalyst has been inferred from XPS data [71,72]. Moreover, reducible metal oxides, such as ZnO, can modify the electronic structure through alloy formation, the formation of Cu^+^ sites, or the creation of new interfacial sites, which enhance the hydrogenation of C=O and C–OH species on Cu [60]. The formation of Cu^+^ species can also generate acid sites, which favor the hydrogenolysis reaction, leading to a higher proportion of MF [71].

In order to increase the dispersion of Cu^0^ nanoparticles on metal oxides, the synthesis of layered double hydroxides (LDHs) or hydrotalcites, which are prepared from M^2+^ (Cu^2+^, Zn^2+^, Mg^2+^, Ca^2+^, Co^2+^) and M^3+^ (Al^3+^, Cr^3+^) species in basic medium, has been proposed. Thus, it has been found that the incorporation of a lower Al_2_O_3_ content (<10%) has a beneficial effect on the catalytic behavior, since Al_2_O_3_ improves the thermal stability of catalysts instead of being an electronic additive. However, the incorporation of larger proportion of Al_2_O_3_ weakens the Cu–ZnO interaction, which is associated with a decrease in the conversion as a consequence of FUR polymerization [73,74].

On the other hand, a positive effect on the catalytic behavior was observed when CeO_2_ was used to disperse Cu species, due to that ceria generates O vacancies [75,76]. In these systems, the presence of a high proportion of interfacial sites between CuO/CeO_2_ enhances the reducibility of the catalytic system, with reduction temperatures very close to the reaction temperature. However, this fact barely affects the deactivation by sintering, as was inferred from XPS, but, rather, is attributed to the formation of carbonaceous deposits, as occurs in Cu/SiO_2_, Cu/MgO, and Cu/ZnO catalysts. With regard to the selectivity pattern, the coexistence of FOL and MF as main reaction products is observed, so Cu/CeO_2_ catalysts also possess acid sites that favor the hydrogenolysis of FOL to MF [75]. In this sense, Jackson et al., reported that CeO_2_ sites favor the ketonization reaction, while the Cu sites are involved in FUR hydrogenation [76]. Table 1 gathers the experimental conditions and catalytic performance in gas-phase hydrogenation of FUR to FOL and MF of different Cu-based catalysts.

On the other hand, Jiménez-Gómez et al., reported that Cu species can be dispersed in inexpensive clay minerals, such as bentonite, sepiolite, or kerolite [79]. It was also confirmed that the acidity of clay minerals is a key parameter in FUR conversion, since the Al-rich clay minerals, i.e., bentonite, present an activity poorer than that of catalysts supported in Mg-rich clay minerals (sepiolite and kerolite). The incorporation of basic or amphoteric oxides, such as MgO, ZnO, and CeO_2_, improves the FOL yield as a consequence of the increase in the amount of available Cu sites, as well as by modification of the electronic density of active phase. Thus, these metal oxides weaken the interaction of FUR molecules with active centers, which favors its desorption, maintaining their availability along the TOS.

Simulation studies have provided an alternative interpretation for the role of these promoters during the hydrogenation of ketones, because reducible metal oxides, such as ZnO and CeO_x_, seems to facilitate water activation on Cu surfaces [60].

The liquid-phase FUR hydrogenation has also shown excellent catalytic results, although higher hydrogen pressures are required compared to the gas-phase process. FUR reduction can be performed by using molecular hydrogen as reducing agent, for which several noble metals have been employed as active phase. However, noticeable drawbacks, such as poor selectivity due to undesired side reactions and high cost of the catalyst as a consequence of the limited abundance of precious metals, are important barriers for large-scale applications. Considering these premises, the scientific community is developing more inexpensive catalysts, such as Cu-based catalysts, to achieve most competitive processes for industrial implementation. 

In this context, Dumesic’s group performed a study by using in operando X-ray absorption spectroscopy with Cu/γ-Al_2_O_3_ catalysts, with observed changes in the catalytic activity being associated with structural modifications of the catalyst [84]. They achieved minimization of the loss of available Cu sites due to sintering, by atomic layer deposition of an alumina overcoat. In addition, Cu nanoparticles also tend to suffer deactivation by the deposition of carbonaceous species, which cover Cu sites. Nonetheless, these type of deactivated active sites can be regenerated by calcination, although this treatment can also be associated with sintering.

Other authors have reported high FOL yields with Cu/Ni/Mg/Al catalysts obtained from hydrotalcites, although at lower H_2_ pressures [85].

Cu species can also play a promoter role in the catalytic behavior of Pd/Al_2_O_3_. Thus, Pd/Al_2_O_3_ mainly produces FOL, which is further reduced to tetrahydrofurfuryl alcohol (THFOL), probably due to the activation of the H_2_ on the Pd surface, generating active H species for the reduction of the C=C bonds of the furan ring. The incorporation of Cu strongly affects the selectivity pattern, obtaining FOL as main product, since the hydrogenating capacity of the catalyst decreases [86].

In summary, the main advantage of the use of Cu-based catalysts is related to the specific interaction of the Cu sites with the carbonyl group of FUR in such a way that the range of products is limited to FOL or MF. However, other catalysts with higher hydrogenating capacity, such as Ni-based catalyst, can interact with the furanic ring and the carbonyl group, which extends the number of products. Regarding the possible reactions, the first step (FUR → FOL), which follows the Langmuir–Hinshelwood reaction, requires higher adsorption heat than the second step (FOL → MF). In the same way, the adsorption heat of FUR and FOL are much higher than MF in such a way that FUR and/or FOL are prone to interact with the support strongly, causing the blockage of the active sites.

### 2.2. Hydrogenation of Furfural through Catalytic Transfer Hydrogenation

**Scheme 2:**
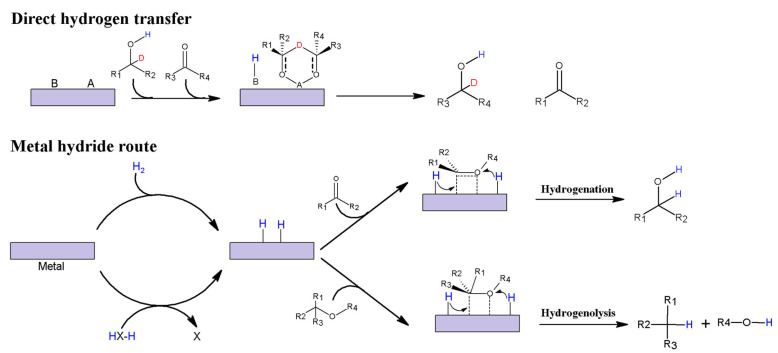
Catalytic transfer hydrogenation of aldehydes by the direct hydrogen transfer (MPV) or metal hydride route.

Some organic molecules, including alcohols, organic acids, or hydrazine, can also act as hydrogen donors instead of exogenous H_2_, through a pathway denoted as catalytic transfer hydrogenation (CTH). The use of these hydrogen donors shows several advantages related to the hydrogen storage, safety, and transportation. In addition, the lower hydrogenating capability of these hydrogen donors diminishes the uncontrolled reactions in hydrogenation and/or hydrogenolysis processes. The CTH reaction can proceed via direct hydrogen transfer through the Meerwein–Ponndorf–Verley (MPV) reaction, or via a metal hydride route. In the case of Cu-based catalysts, the CTH process follows the second pathway (Scheme 2) [4]. In Table 2, there is a list of the most noticeable catalytic systems developed for the reduction of FUR to FOL through CTH reaction.

Several transition metals, such as Ru, Pt, and Cu, are able to activate H–H, C–H, C=O, or C–O bonds. Among them, Cu-based catalysts gained a high interest since they exhibit exceptional activity in the cleavage of C–O bonds, while the noble metal catalysts, or Ni-based catalysts, tend to hydrogenate the ring or favor the decarbonylation [87]. Commonly, these catalysts are present as small metal nanoparticles, which are dispersed on the surface of thermally stable supports with high specific surface area, such as alumina [88], zeolites [89], or activated carbons [90], among others. Generally, the first step of the CTH, through the metal hydride route, is the dissociative adsorption of H_2_ into atomic hydrogen, while the metal-catalyzed CTH process starts by the preliminary activation of the organic hydrogen donor, forming negatively charged hydride species [91]. The presence of different H species (atomic hydrogen and hydride) seems to indicate substantial mechanistic differences between the use of H_2_ and organic hydrogen donors [4].

**Table 2:** Catalytic activity of Cu-based catalysts for conversion of FUR to FOL through CTH.

Catalyst	Hydrogen Source	T (°C)	P (MPa)	t (h)	C ^a^ (%)	Y_FOL_^b^ (%)	Ref.
Cu/Fe_2_O_3_	Isopropanol	180	0.1	7.5	37.0	28.0	[87]
Cu/Ac-SO_3_H	Isopropanol	105	0.4	2	>99.9	>99.9	[92]
Co–Cu/SBA-15	Isopropanol	170	2	4	80.1	79.6	[93]
Co–Cu/SBA-15	Isopropanol	130	4	3	-	94.8	[94]
Cu_1_Co_5_	Isopropanol	180	2	5	100	38.1	[95]
Cu/MgO–Al_2_O_3_	Isopropanol	210	-	1	>99.9	89.3	[96]
Cu/SiO_2_	Isopropanol	110	1	4	66.3	66.3	[97]
CuZnAl	Isopropanol	110	1	4	48.1	48.1	[97]
CuMgAl	Isopropanol	110	1	4	100	100	[97]
CuCr	Isopropanol	110	1	4	93.2	93.2	[97]
CuMgAl	Isopropanol	150	1	6	100	100	[98]
Cu/ZnO–Cr_2_O_3_-ZrO_2_	Isopropanol	170	2	3.5	100	96	[99]
CuNi_2.5_@C	H_2_O	130	5	5	71.6	16.7	[100]
Cu/ZrO_2_	Isopropanol	220	0.1	4	98.9	38.7	[101]
Cu–Ru/ZrO_2_	Isopropanol	220	0.1	4	100	36.3	[101]
Cu–Ni/ZrO_2_	Isopropanol	220	0.1	4	100	33.7	[101]
Cu@Pt	Isopropanol	250	0.69	1.5	15.3	13.8	[102]
Cu–Ni/Al_2_O_3_	Isopropanol	190	0.1	4	90	54	[103]
Cu–Ni (bulk)	Decanol	130	5	6	39	39	[104]
Cu/Al_2_O_3_	Methanol	245	1	1.5	>99	540	[105]
Cu–Pd/C	1,4-dioxane	170	0.1	3	100	98.1	[106]
Cu/MgAl_2_O_4_	Formic acid	210	-	1	90.0	89.1	[107]
Cu–Ni/γ-Al_2_O_3_^c^	Isopropanol	130	4	4	92.6	86.7	[108]
NiCu/Al_2_O_3_	Isopropanol	200	0.5	2	>99.9	50.0	[109]
CoCu/Al_2_O_3_	Isopropanol	200	0.5	2	>99.9	63.0	[109]

^a^ C: furfural conversion; ^b^ Y_FOL_: furfuryl alcohol yield; ^c^ Cu:Ni molar ratio of 1.

As previously indicated, alcohols are largely used as hydrogen donors in CTH processes. Among them, secondary alcohols are better hydrogen donors than primary alcohols over metallic surfaces. The easier diffusion of H species in secondary alcohols is ascribed to the alkyl groups stabilizing the intermediate reaction, due to the inductive electron donation to the α–C of the alcohol [110,111]. In this sense, Vlachos et al., pointed out that a longer alkyl chain in the alcohol displays a beneficial effect in the CTH reaction [110].

Scholtz et al., carried out the FUR reduction over metals (Pd, Ni, and Cu) supported on Fe_2_O_3_ and using 2-propanol as a hydrogen donor, reaching the highest activity with Pd/Fe_2_O_3_, although the most selective catalyst was Cu/Fe_2_O_3_, with FOL as main product, since Cu sites are only coordinated with the carbonyl group in a η^1^(O)-aldehyde conformation [87].

Gong et al., proposed another catalytic system where Cu^0^ species were dispersed on an active carbon functionalized with sulfonic groups [92]. They established that –SO_3_H groups can play two roles, since, on one hand, they can favor the stabilization and dispersion of Cu particles and, on the other hand, they can strengthen the adsorption of FUR molecules by hydrogen bonds, promoting FUR hydrogenation. However, this strong interaction implies that a high reaction temperature and/or pressure is required to obtain FOL. Moreover, the presence of unreduced Cu^+^ species can also generate Lewis acid sites, which can also activate the carbonyl group through the electron lone pair of oxygen.

In other studies, Srivastava et al., indicated that the incorporation of Co as a promoter can facilitate the dispersion of Cu species, as well as favor their reduction [93,94], while the presence of unreduced Cu^+^ and Co^2+^ species generates Lewis acid sites, and stronger reaction conditions favor the hydrogenolysis reaction to form MF [95]. Kinetic studies have reported that the reaction follows a pseudo-first-order kinetic with respect to FUR in liquid-phase reaction [94].

Similarly, several authors have synthesized Cu/MgO/Al_2_O_3_ and Cu/ZnO/Al_2_O_3_ catalysts from hydrotalcites, which were highly active and selective to FOL [96,97,98]. According to Gilkey et al., these catalytic systems can provide H species by two ways [4]. Thus, both the secondary alcohol and FUR can be adsorbed on the Lewis acid sites of MgO/Al_2_O_3_, where a hydrogen located in the α-position of the alcohol is transferred to the carbonyl group of FUR through a six-membered ring intermediate to form FOL. Likewise, the H species can be provided from Cu-mediated hydrogenation, since the H atoms can be also adsorbed and transferred on these metallic sites [96]. In this sense, it has been reported that the dispersion, and, consequently, the amount of available Cu sites can exert an important effect on the catalytic behavior in liquid-phase FUR hydrogenation [97]. Cu:Zn:Cr:Zr-based catalysts display a similar behavior, where both Zn and Cr species favor the dispersion and reducibility of Cu species to create metallic sites, while ZrO_2_ provides Lewis sites, which can be involved in the hydride transfer via the MPV reaction [99].

On the other hand, Wang et al., developed Cu@C and CuNi@C catalysts from MOFs [100]. The incorporation of Ni species improves the catalytic performance, especially the conversion of 2-cyclopentenone (2-CPE) to cyclopentanone (CPO), which takes place in several steps. Firstly, FUR is hydrogenated to FOL, influenced by several parameters, such as H_2_ pressure, reaction temperature, Cu:Ni molar ratio, and dispersion of the active phase. Then, it requires H^+^ species for the rearrangement reaction of FOL to 2-CPE, which suffers a total ring hydrogenation to form CPO. Finally, CPO can be reduced to cyclopentanol (CPL), although more severe experimental conditions, or longer reaction times, are required.

Chang et al., attained an MF+MTHF yield close to 84% using Cu–Pd catalysts and 2-propanol as hydrogen donor [101]. In the same way, the selectivity of Pt catalysts was modulated by the incorporation of Cu species, since the presence of Cu weakens the binding strength of H_2_ on noble metal surface, minimizing the blockage of these noble metal sites, as a consequence of the strong interaction with FUR molecules, and modulates the selectivity towards FOL [102].

CuNi-based catalysts have also been studied in FUR hydrogenation by CTH, obtaining, with a CuNi_2_Al catalyst, a maximum MF yield of 65% at 230 °C and an MTHF yield of 51% at 250 °C [103]. Other authors have carried out studies at milder reaction temperatures with CuNi, obtaining a greater selectivity towards FOL, in such a way that the reaction temperature again seems to be a key factor that determines the selectivity pattern [104]. These authors also reported that the use of lower reduction temperature causes a partial reduction of Cu, and, mainly, of Ni species, which also favor side reactions between FUR and 2-propanol to form i-propyl furfuryl ether, that, notwithstanding, can be used as fuel additive.

Methanol has also been used as hydrogen donor, since its dehydrogenation yields H and formaldehyde. In turn, the latter can react with H_2_O molecules to generate formic acid, which can decompose in CO_2_, and more H species. In this way, the number of H species formed from methanol is twice that obtained from secondary alcohols [19]. 

Focusing on Cu-based catalysts, Zhang et al., reported the CTH of FUR with methanol as hydrogen donor, using inexpensive hydrotalcite as precursors to disperse Cu species, reaching a high MF yield [105]. They pointed out that methanol steam reforming takes place on CuO species, providing H_2_, as well as CO and CO_2_, as byproducts. On the other hand, dimethylether and H_2_O molecules can be obtained from the etherification of methanol as secondary reaction. In the next step, Cu^2+^ species are reduced to Cu^+^ and Cu^0^ with H_2_ molecules. In turn, these Cu^0^ species also promote methanol steam reforming to produce a greater amount of H_2_. Then, as was indicated by other authors, H species, obtained from a homolytic cleavage, only attack the carbonyl group through a hydroxyalkyl intermediate to form FOL [56]. The presence of acid/basic sites can favor secondary reactions between FOL and methanol, leading to hemiacetalization, acetalization, or aldol reactions. In a final step, FOL is protonated by an H on the Cu surface. Subsequently, C–O bond at the α-position was dissociated to form an H_2_O molecule in the presence of Lewis acid sites and, then, another hydrogen atom attacks the carbon in α-position on the Cu surface to form MF [105].

On the other hand, formic acid is another compound that can be considered as a hydrogen donor. Formic acid can be obtained as a product in the fast pyrolysis of lignocellulosic biomass, so it is an environmentally friendly source to produce hydrogen donor for CTH reactions [23,112,113]. The use of formic acid can favor the adsorption of hydrogen species, as well as the adsorption of formate species.

In order to cheapen the noble-metal-based catalysts, Pd–Cu/CuO/MgO catalysts have been synthesized, reaching an FOL yield above 98% [106]. It was hypothesized that CuO is dissolved and, then, Cu^2+^ species are reduced by H generated from the formic acid, leading to Cu^0^ nanoparticles that are deposited on the Pd/MgO catalyst. The incorporation of Cu^0^ species limits the over-reduction associated with Pd active sites, since it favors the selective hydrogenation of the carbonyl group, because FUR molecules are adsorbed on unreduced Cu^2+^ species through the oxygen atom of the carbonyl group.

As previously indicated, Cu/MgO–Al_2_O_3_ reduces FUR to FOL in the presence of formic acid, as H_2_ is obtained from the decomposition of formic acid (HCOOH → H_2_ + CO_2_), which, in turn, suffers a homolytic rupture on Cu sites. Then, these H species only attack the carbonyl group of FUR [107].

### 2.3. Hydrogenolysis of Furfural to 2-Methylfuran

The catalytic C–O hydrogenolysis plays an important role in biorenewable upgrading strategies, and many mechanisms have been proposed as a function of the catalyst, reactant, and reaction conditions. A reaction system that involves C–O hydrogenolysis is the conversion of FUR to MF. FUR has been demonstrated to be a renewable and versatile platform molecule for the synthesis of chemicals and fuels [3,4]. Many efforts have recently been made in the FUR hydrodeoxygenation (HDO) reaction, due to combustion performance in engines of MF, that can be directly used as a biofuel, due to its octane number of 74, or it can be further transformed into high-alkane liquid fuels through condensation. Moreover, MF can be used as a solvent and chemical intermediate for the production of antimalarial drugs (chloroquine), chrysanthemate pesticides, fragrances, MTHF, and nitrogen and sulfur heterocycles [114,115,116,117,118]. The commercial production of MF from FUR has the problem of side reactions that lead to the formation of many products, such as tetrahydrofurfuryl alcohol (THFA) from FOL, MTHF and 2-pentanol from hydrogenolysis of THFA, and furan from the decarbonylation of FUR [119,120].

Ricard and Guinot disclosed a procedure for the manufacture of FOL and MF via gas-phase hydrogenation of FUR [46]. They used a reduced copper oxide catalyst on different backing substances (asbestos, pumice stone, silica, and kaolin), but, at 140 °C, the percentage of MF was only 10–20% of the FOL formed. Moreover, the catalyst could be regenerated by passing hot air through it and performing further reduction. By using a pure copper obtained by reducing copper oxide precipitated on inert supports, such as asbestos, silica, and pumice, it was possible to increase the MF yield from 40 to 100% by feeding FOL instead of FUR, at 170 °C [121]. No resins or byproducts were detected, since the large amount of heat evolved in the hydrogenation process was rapidly removed.

On the other hand, copper chromite dispersed on activated lump charcoal was revealed as a very stable catalyst for the synthesis of MF from FUR in gas phase at temperatures of 200–225 °C [122]. MF was purified by distillation, attaining a yield of 95%. The catalyst was used intermittently over a period of three months, with an overall yield for this period of 91%, with a weight of MF produced that was 23 times that of the catalyst used.

In the last few years, many catalysts have been developed for the production of MF from biomass-based FUR in gas and liquid phase. Cu-containing catalysts, such as Raney Cu, Cu/Al_2_O_3_, and copper chromite, have been studied [50,123,124], but they were found to quickly deactivate. The main cause of deactivation of these catalysts is poisoning due to strong adsorption of polymeric species formed during reaction. For the copper chromite catalyst, a Cr coverage of Cu sites was also reported as an additional cause of deactivation at high temperature [51]. Moreover, the toxic nature of chromium greatly restricts its practical application. Therefore, the development of nontoxic catalysts capable of selectively converting FUR to MF is an important challenge today. Many publications have demonstrated that metal catalysts are efficient for the selective removal of oxygen from the carbonyl group under mild experimental conditions. HDO catalysts are mainly based on very expensive precious metals, and, among these, the most efficient have been those based on Pd, with MF yields higher than 90%, although high reaction temperatures are required for HDO [125,126], and Ru, in which Lewis acid sites are necessary to obtain MF from FUR [110,122].

An attractive approach is the use of catalysts based on non-noble metals. In this sense, Cu-based catalysts have been demonstrated to be active in this reaction, either in vapor as liquid phase. These are less oxophilic than that based on group 8 elements, so they interact weakly with the furan ring, forming FOL, by hydrogenation of the carbonyl of FUR, followed, to a lesser extent, by hydrogenolysis to MF. This lower activity in the C–O hydrogenolysis of Cu-based catalysts was primarily compensated by increasing reaction temperature (ca. >250 °C) [127]. The addition of an oxyphilic metal and/or the presence of Lewis acid sites are other options, hardly studied, with the aim of increasing the production of MF from FUR with Cu-based catalysts. In this section, we review the most relevant studies concerning the production of MF from FUR on Cu-based catalysts carried out both in gas and in liquid phase.

#### 2.3.1. Gas-Phase Hydrogenolysis of Furfural to 2-Methylfurane 

Many authors have studied the catalytic behavior of monometallic copper-based catalysts in this reaction. Thus, Sitthisa et al. [56] compared the catalytic performance of Cu, Pd, and Ni supported on SiO_2_ in the HDO of FUR in a continuous-flow reactor, under atmospheric pressure of hydrogen, between 210 and 290 °C. They found different product distribution depending on the metal nature, due to the strength of interaction of the furan ring with the metallic surface, that generated different reaction intermediates. Cu-based catalyst produced mainly FOL, while Pd and Ni yielded decarbonylation products and Ni also formed ring-opening products, due to its stronger interaction with the furan ring. These results show that Cu-based catalysts have a preferred adsorption mode *η*^1^(O)-aldehyde, and a very weak interaction with C=C bonds. A computational study on the reaction mechanism also revealed this preferred adsorption on the Cu (111) [58]. Thus, for FOL formation from FUR, this study concluded that an H atom is firstly added to the C of the C=O group, but the rate-determining step is the addition of the second H to form FOL. For MF formation from FOL, water formation is the rate-determining step. By studying the reaction mechanism via DFT calculations combined with experimental data obtained with Cu/ZnO catalysts, it was also concluded that MF is easily formed and shows a low adsorption energy, while the H_2_O formation needs to overcome a high surface barrier. Therefore, the removal of the hydroxyl group is difficult, which could be unfavorable for the conversion of FOL to MF [128]. Furthermore, the elucidation of reaction rates at different temperatures showed the preferential formation of MF at high temperature, as the reaction intermediate (FOL) was consumed.

**Figure 3:**
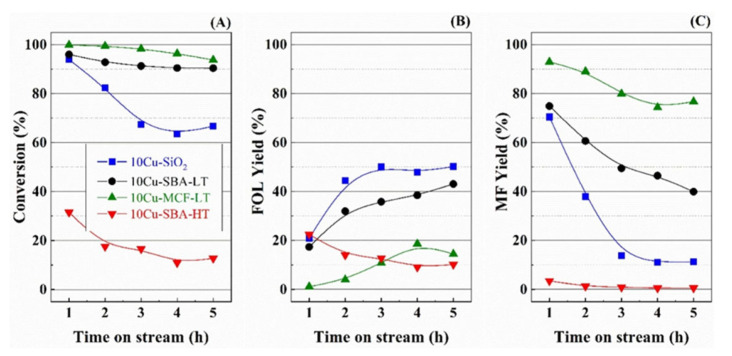
FUR conversion (**A**), FOL yield (**B**), and MF yield (**C**) in the FUR hydrogenation over 10Cu–Y catalysts. Reprinted with permission from [130]. Copyright 2022 Elsevier.

Among the options mentioned above to improve the productivity to MF from FUR by using Cu-based catalysts, the nature of support is a crucial parameter due to the presence of acid sites that can be catalytically effective centers in the hydrogenolysis of the C–O bond, and also produce metal–support interactions that influence the nature of the supported species. In this way, Cu-based catalysts supported on SiO_2_, Al_2_O_3_, and ZnO were explored for the production of MF, with the aim to study the influence of the acid–base properties of the support on product distribution [61]. The best performance was obtained for Cu/SiO_2_ catalyst, with 89.5% yield to MF, at 220 °C and atmospheric pressure. The synergistic effect between Cu and weak acid sites generated highly dispersed Cu nanoparticles, which activated the CH=O group of FUR and dissociated the CH_2_–OH group of the intermediate FOL, as the authors demonstrated by temperature-programmed desorption of FUR. Recently, a Cu/SiO_2_ catalyst prepared by the ammonia evaporation method was shown to be more effective for the production of MF than that prepared by co-precipitation [129]. The high selectivity toward MF (80% with almost complete conversion of FUR) was related to the high dispersion of Cu nanoparticles and the formation of a large content of Cu^+^, which enhances oxophilicity and acts as weak acid sites. Similar conclusions were proposed by Jiménez-Gómez et al. [130], who recently studied the dispersion of Cu nanoparticles on several porous silicas (commercial fumed silica 10Cu–SiO_2_, SBA-15 synthesized at room temperature (10Cu–SBA-LT) and under hydrothermal conditions (10Cu–SBA-HT), and mesocellular foam). They demonstrated that catalytic behavior was highly dependent on the acidity, morphology, and textural properties of the support. Thus, the addition of fluoride ions during the synthesis of SBA-15 shortened the length of silica channels (10Cu-MCF-LT), giving rise to catalysts with a higher metallic surface area and lower acidity, thus providing high FUR conversion (95%) and MF yield (76%) at 190 °C after 5 h of reaction (Figure 3). 

For longer reaction times, the catalyst became more selective to FOL, due to the formation of carbonaceous deposits on the acid centers responsible for the hydrogenolysis of FOL to MF. On the other hand, Al_2_O_3_ is one of the most used supports due to its large surface area and moderate acidity. In this sense, a mesoporous Cu/Al_2_O_3_ catalyst, prepared by deficient solvent precipitation, showed quite promising results in furfural HDO, due to improved Cu accessibility and the coexistence of Cu^0^ and Cu^+^ states [131]. This catalyst also exhibited a high stability due to the strong metal–support interaction. The calcination temperature was optimized to avoid the formation of spinel structures, which decreased the amount of Cu active sites, porosity, and acidity. The FUR activity was 31.9 mmol g_cat_^−^^1^ h^−^^1^ with an MF selectivity of ca. 80%, at 190 °C, WHSV = 23 h^−^^1^, and H_2_:FUR molar ratio = 20. 

Yang et al. [71] studied Cu-based catalyst supported on two different mineral-derived ZnO (aurichalcite and zincian malachite). The better performance of the aurichalcite-derived catalyst in the production of MF (94.5% yield at 200 °C) was also attributed to its surface acidity, the higher dispersion of copper species, and the stronger Cu^0^–ZnO synergistic effect. The use of other supports, such as CeO_2_, also revealed that this strong interaction with copper species would allow to obtain highly efficient and stable catalysts for HDO of FUR [75]. The presence of CeO_2_ favored the reduction of copper oxide at lower temperature, which was a key factor to obtain smaller Cu^0^ particles, minimizing the sintering effect. MF was the main product at short reaction time, and, along time-on-stream, the selectivity to FOL increased due to the formation of carbonaceous deposits on the highly hydrogenating sites and the progressive oxidation of Cu^0^ by water generated in the HDO process. The average Cu^0^ crystallite size is also an important factor in this catalytic reaction, to the extent that small nanoparticles favor the formation of MF, as reported by Jiménez-Gómez et al. [62] for a series of copper supported on mesoporous silica materials synthesized by complexation with the amine group of dodecylamine, as structure-directing agent, to obtain high dispersion of precursor Cu^2+^ species. A yield of 95% to MF was obtained for the catalyst with 10 wt.% of copper, after 5 h of reaction at 210 °C, but this low particle size simultaneously generated a strong deactivation due to the adsorption of FUR or FOL on the active sites, forming coke and blocking the active centers.

Another promising strategy to improve the productivity of MF from FUR by using Cu-based catalysts is the use of bimetallic catalysts, because the second metal can modify the catalytic properties of Cu due to structural and/or electronic effects. This second metal can create active sites with an weaker interaction between the active site and furan ring, which improves selectivity towards MF. In this sense, the use of Fe as promoter in Cu-based catalysts has been investigated by different research groups. Thus, Lessard et al. [132] studied a CuFe/SiO_2_ catalyst which achieved the selective HDO of FUR to MF with a 98% yield, but with a marked deactivation after 20 h. The addition of Fe to a silica-supported Cu catalyst increased the selectivity toward MF (over 90%). In these catalysts, active sites were generated after reduction at 270 °C, at which Cu was fully reduced, whereas Fe was only partially reduced, and a mixture of Fe^3+^ and Fe^2+^ was observed. These iron species were responsible for the increase in the production of MF, because the partially reduced iron species favor the selective conversion of FOL to MF [133]. NiCu-based catalysts have been demonstrated to be another effective bimetallic system for HDO. Xiong et al. [134] explored the effect of bimetallic formation on enhancing the HDO activity by studying Cu (111) and Ni/Cu(111) surfaces using DFT calculations, temperature-programmed desorption (TPD), and high-resolution electron energy loss spectroscopy (HREELS). These studies pointed out an increased interaction with the carbonyl group of FUR for NiCu bimetallic surfaces compared with that on Cu(111). Bimetallic CoCu-based catalysts with a high metal content (48–63 wt%) were obtained by high-temperature pyrolysis of CoCu/MOF (metal−organic framework). Both composition and pyrolysis temperature affected the catalytic performance. The higher catalytic rates and increased selectivity toward to MF were obtained for high Co:Cu ratios and pyrolysis temperatures lower than 600 °C, because smaller nanoparticles with a less complete Cu shell with more exposed Co were formed [135]. A significant improvement in the catalytic production of MF from FUR was achieved by using nanoporous Cu–Al–Co ternary alloy catalysts [136]. In particular, ~5 atomic % Co content showed 98.2% overall conversion with 66% MF selectivity at 240 °C. Moreover, these catalysts were stable under the experimental conditions used in this study, as confirmed by XAS studies. 

In this section, it was shown that the production of MF from FUR highly depends on the acidity, morphology, and textural properties of the support. The acid sites are catalytically effective centers in the hydrogenolysis of the C–O bond and also generate highly dispersed Cu nanoparticles. The Cu–support interaction originates high metallic dispersion and the presence of Cu^+^, which favors the formation of MF. In addition, the use of bimetallic catalysts improves the selectivity towards MF because the incorporation of a second metal causes structural and/or electronic changes in Cu, resulting in more efficient and stable catalysts for the selective production of MF from FUR.

#### 2.3.2. Liquid-Phase Hydrogenolysis of Furfural to 2-Methylfurane 

High reaction temperatures and high H_2_ pressures are needed to carry out the gas-phase hydrogenolysis of FUR. Moreover, the reaction is often accompanied by catalyst deactivation due to coke deposition, requiring cycles of calcination to remove the deposited coke, and reduction to regenerate the metallic species. To avoid these problems, many catalysts have been developed to synthesize MF via liquid-phase FUR HDO [2,137,138,139]. Moreover, liquid-phase hydrogenation is most likely preferred for compatibility with the upstream production of FUR [140]. Many studies have been developed for this reaction in liquid phase by using Cu-based catalysts as both mono- and bimetallic systems. Monometallic Cu-based catalysts have provided low yields to MF due to both the weak adsorption of FUR and the low dissociation of H_2_ on copper surface. Thus, bimetallic catalysts are preferred, due to a better performance in liquid phase. In addition, many studies were focused on the addition of a metal oxide as promoter. 

Xu et al. [85] found that the Cu particle size highly affects the selectivity to MF of CuNiMgAl catalysts prepared from hydrotalcite-like precursors. They observed a decrease in both FUR conversion and FOL selectivity upon increasing the activation temperature, due to the agglomeration of Cu^0^ particles, because larger particles favored FOL hydrogenolysis to MF. Concerning bimetallic catalysts, a series of Cu–Fe catalysts, synthesized from metal nitrates, exhibited highly efficient performance in HDO of FUR, producing 51% yield of MF under mild conditions [141]. These authors found that the reaction temperature had an important influence on the product distribution; thus, the yield to MF increased with the reaction temperature (at 200 °C, 51.1% MF and 41.7% FOL were obtained), but FUR polymerization took place at higher temperatures. 

Similar to other studies previously mentioned, Srivastava et al. [108,137,142] explored the roles of support acidity and metal–support interaction, on which depend reducibility, metal particle size, and metal dispersion. They studied the combination of Cu^0^ and CoO_x_ in the production of MF via hydrogenolysis of FUR in liquid phase. The presence of metallic Cu and partially reduced cobalt species can break the C–O bond. For bimetallic Cu–Co catalysts supported on SiO_2_, H-ZSM-5, and *γ*-Al_2_O_3_, the properties of carriers and the strong Cu–Co interaction had important influence on the catalytic performance; thus, the order of the selectivity towards MF was: Cu–Co/*γ*-Al_2_O_3_ > Cu–Co/H-ZSM-5 > Cu–Co/SiO_2_, the best performance being for the CuCo/*γ*-Al_2_O_3_ catalyst with 78% yield to 2-MF, at 220 °C and 4 MPa [137]. Srivastava et al., also reported optimization and kinetics study along with mechanism for CuCo/Al_2_O_3_ catalyst in this reaction [108]. The mechanism for the hydrogenolysis of FUR to MF on Cu-based catalysts in liquid phase is not fully understood. They concluded that MF can be produced via C=O hydrogenation over Cu^0^, due to a preferential adsorption of FUR on Cu^0^ surface, and then acid sites catalyze the hydrogenolysis of FOL to MF. The same synergistic effect between Cu and Co species was recently proposed for a series of highly ordered mesoporous CuCo oxide catalysts, synthesized by the nanocasting method, using mesoporous KIT-6 silica as template [95]. These catalysts were shown to be much more active than a physical mixture of mesoporous Co_3_O_4_ and CuO with the same Cu/Co ratio. The production of MF increased with the decrease in the Cu/Co ratio, and reaction pathway was demonstrated to follow FUR hydrogenation to FOL and, then, hydrogenolysis to MF, as previously observed by other authors. Akmaz et al. [143] also reported a synergistic effect between Cu and Co on MF selectivity in HDO of FU with CoCu/ZrO_2_ catalysts prepared by a sol–gel method. The increase in the amount of Co in the catalyst facilitated hydrogenation of FOL to MF, but an excess of Co provoked the hydrogenation of MF to MTHF. The highest MF yield was 92.2% at 100% FUR conversion, at 200 °C for 4 h, under 1.5 MPa H_2_. The catalyst showed good reusability, because after five catalytic cycles, MF yield did not change significantly.

The metal–metal oxide synergistic effect was also observed in monometallic catalysts between Cu^0^ and copper oxide [92]. Monometallic copper supported on an activated carbon presented uniform sized Cu nanoparticles, with the adequate Cu^2+^:Cu^+^:Cu^0^ ratio, to transform FUR into MF with almost 100% yield after 4 h, at 170 °C, and 3 MPa H_2_ pressure. Mechanistic studies revealed the formation, in a first step, of FOL at low reaction temperature (80–130 °C) and the hydrogenolysis of the C–O bond to give MF at high reaction temperature (140–170 °C). Moreover, this catalyst was stable and recyclable with only a slight decrease in MF yield from the third run. Geng et al. [144] recently studied a catalyst based on Cu anchored on N-doped reduced graphene oxide, which also showed a high performance due to the synergistic effect between Cu^0^ and Cu^+^ species on the catalyst surface. The yield of MF reached 95.5%, with a 100% FUR conversion, at 240 °C and 1.5 MPa H_2_, after only 3 h of reaction. Moreover, this catalyst possessed high catalytic stability without deactivation after five cycles of reaction. That is why the authors proposed it as a promising catalyst for the sustainable process scaling-up. 

The comparison of the catalytic behavior of mono- and bimetallic catalysts evidences the synergistic effect when two metals interact with the catalyst surface. In this sense, Jaatinen et al. [145] studied the liquid-phase FUR hydrogenation with mono- and bimetallic Cu, Ni, and Fe catalysts supported on activated carbon. Each metal led to a different product distribution and, although monometallic Cu-based catalyst resulted in low activity, the bimetallic combinations with Ni or Fe were observed to decrease further reactions of the desired product, MF, decreasing the formation of products such as MTHF and 2-pentanone. Moreover, they achieved the highest yield for MF (48.9%) with Ni/C catalysts in only 2 h, compared with other studies, where similar yields were obtained at longer reaction time (5–14 h) [140,141]. Another study, also based on bimetallic catalysts, analyzed the addition of molybdenum to a NiCu/SiO_2_ catalyst, which originated the formation of a NiMo(Cu) solid solution with Mo^x+^ species on the catalyst surface, that increased the activity in FUR hydrogenation, improving MF yield [146]. It was shown that an increase in reaction temperature also increased MF yield and led to the complete hydrogenation. At low temperatures, a small amount of MF was formed, the main products being FOL and THFA.

**Figure 4:**
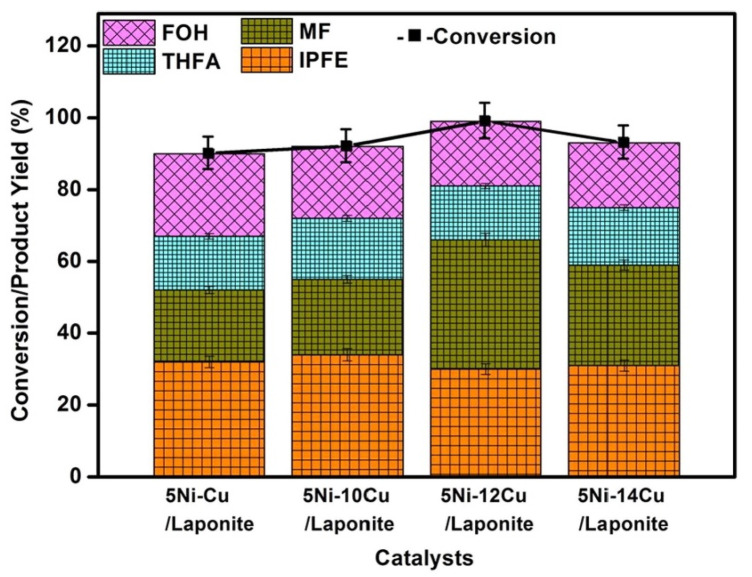
Furfural conversion and product yield over the catalysts at 190 °C for 4 h under 20 bar H_2_ pressure (FOH = furfuryl alcohol, IPFE = isopropyl furfuryl ether). Reprinted with permission from [149]. Copyright 2022 Elsevier 221.

Not only the addition of promoters, but also the support itself, can control the adequate surface structure and composition of bimetallic catalysts. Thus, Bhogeswararao et al. [147] recently studied a bimetallic CuNi catalyst supported on TiO_2_ and Al_2_O_3_ and established that the nature of the support exerts an important effect on the structure of bimetallic CuNi particles and, consequently, on the catalytic activity, selectivity, and stability for FUR conversion to MF. Nevertheless, Cu segregation was observed when TiO_2_ was used as support, due to the strong interaction between Ni and TiO_2_, while a homogeneous distribution of both metals was obtained when Al_2_O_3_ was used. These different compositional structures originated the production of MF when TiO_2_ was used as support, while FOL and THFA were mainly obtained for Al_2_O_3_-based catalysts. The combination of Cu and Ni was also selected to obtain CuNi catalysts supported on carbon foams from tannic acid and from pine bark extracts [148]. Washing the active carbon foam with acid increased the surface oxygen content, which gave rise to a higher selectivity towards MF. The batch hydrotreatment of FUR (230 °C, 4 MPa H_2_) by using CuNi supported on pine-bark-extract-based activated carbon foam washed with HNO_3_ produced the highest MF selectivity (48%) at a conversion of 91%. Another series of bimetallic NiCu catalysts with different Cu loading, supported on laponite clay, also showed synergy between metal Cu and Ni species in the HDO of FUR under reduced hydrogen pressure in liquid phase [149]. The best catalytic performance was obtained for the catalyst with a 12 wt.% Cu loading due to the high dispersion of copper and the high surface acidity, which were directly related to the availability of a larger number of accessible active sites for the hydrogenation of furfural (Figure 4).

Another recent study demonstrated the promoting effect of adding small amounts of oxyphilic Re species on Cu/*γ*-Al_2_O_3_ catalyst in hydrogenolysis of the C–OH bond in FOL to form MF during the HDO of FUR in liquid phase [150]. An 86% MF yield with 100% FUR conversion was attained at 200 °C for 6 h, with an initial H_2_ pressure of 2 MPa. Moreover, the catalytic performance remained highly stable through four consecutives cycles of reaction. The reaction mechanism proposed involved the carbonyl group of FUR firstly adsorbed on the oxophilic sites and, then, the H atom, resulting from dissociation of H_2_ on metallic Cu and Cu spinel sites, was transferred to hydrogenate carbonyl group to form FOL which was desorbed. The adsorption of the hydroxyl group of FOL was improved by the presence of Re on the Cu species, which favored further hydrogenolysis of FOL to MF.

#### 2.3.3. Conversion of Furfural to 2-Methylfurane through Catalytic Transfer Hydrogenation

Catalytic transfer hydrogenation (CTH) is a selective route that uses organic compounds, such as alcohols or organic acids, as hydrogen donor, representing a greener and low-cost alternative hydrogenation route for the production of MF from FUR without using high hydrogen pressure. Table 3 lists the main catalytic systems applied to this reaction for furfural reduction to MF. CTH studies have also been carried out both in the gas and liquid phase, and many of them have employed noble-metal-based catalysts, such as Pd [87] and Ru [110,140]. In general, these are expensive, with low yields to MF and poor stability under the reaction conditions used. However, Cu-based catalysts have provided higher MF yields in gas-phase CTH [3], and the most relevant results are summarized below.

Li’s group reported several studies of the gas-phase CTH with Cu-based catalysts [117,151,152,153]. They developed a coupling route to obtain *γ*-butyrolactone and MF by simultaneous dehydrogenation of 1,4-butanediol and hydrogenation of furfural in the same reactor. Thus, FUR was reduced by 1,4-butanediol, which is oxidized to *γ*-butyrolactone, producing MF with a yield of 96.5%, by using a CuZn catalyst, at 210 °C and 0.1 MPa H_2_ [116]. The coupling of both reactions was an efficient process compared to conventional FUR hydrogenation, because it was conducted at a lower reaction temperature and without hydrogen supply. Cu–Zn–Al catalysts, which are important industrial catalysts for hydrogenation, have also been studied in this coupling process, resulting that the different calcination temperature has a significant effect on the catalytic performance. Thus, a Cu–Zn–Al catalyst calcined at 350 °C showed an optimum catalytic performance with an MF yield of 93% at 225 °C and 0.1 MPa H_2_ [117]. Under similar experimental conditions, a copper chromite catalyst, prepared by co-precipitation method, showed a conversion of FUR higher than 99.7% and a selectivity toward MF over 94.7% [154].

**Table 3:** Catalytic activity of Cu-based catalysts for conversion of FUR to MF through CTH.

Catalyst	Hydrogen Source	T (°C)	t (h)	C ^a^ (%)	Y_MF_^b^ (%)	Ref.
CuPd/ZrO_2_	Isopropanol	220	4	100	61.9	[101]
Cu–Zn–Al	1,4-butanediol	225	10	99.9	93.0	[117]
Cu–Zn–Al	Cyclohexanol	270	8	99.2	93.0	[152]
Cu–Mn–Si	Cyclohexanol	290	8	99.8	94.0	[153]
Copper–chromite	1,4-butanediol	205	10	99.7	94.3	[154]
Cu–FeO_x_	Isopropanol	190	4	98.0	82.2	[155]
NiCu/Al_2_O_3_	Formic acid	210	4	97.4	75.6	[156]
CuRe/Al_2_O_3_	Isopropanol	220	4	100	94.0	[157]
Cu–Zn–Al	Isopropanol	180	4	96.0	72.0	[158]
Cu/γAl_2_O_3_	Isopropanol	200	2	100	20.7	[109]
CoCu/γ-Al_2_O_3_	Isopropanol	200	2	100	29.0	[109]
NiCu/γ-Al_2_O_3_	Isopropanol	200	2	100	41.1	[109]
Cuº/Cu_2_O·SiO_2_	Methanol	220	2	100	90.0	[159]
CuO	Methanol	240	4	95.0	60.0	[160]
CuFe_2_O_4_	Isopropanol	200	1.5	99.4	97.0	[161]

^a^ C: conversion; ^b^ y_MF_: MF yield.

Another coupling route was proposed for the synthesis of MF and cyclohexanone, simultaneously, in which the hydrogenation of FUR and the dehydrogenation of cyclohexanol were combined [152,153]. The Cu–Zn–Al catalyst, previously mentioned, was also studied in this coupling reaction. The yields of cyclohexanone and MF increased compared with those in the individual processes. The authors explained this catalytic improvement to the generation of activated hydrogen species on the catalyst surface, due to cyclohexanone dehydrogenation, which played an important role in the hydrogenation of FUR to produce MF. Thus, at 270 °C and 0.1 MPa H_2_, the MF yield was 93%. Moreover, this catalyst exhibited a relatively high stability after 200 h on stream [152]. A series of CuMnSi-based catalysts, prepared by co-precipitation, was also shown to be effective in this coupling process. Its catalytic behavior was compared with that of three model catalysts (Cu-free, Si-free, and Mn-free) in order to investigate how Cu–SiO_2_ and Cu–Mn interactions influence the catalytic performance. The addition of Mn to the Cu–Si catalyst enhanced the dehydrogenation of cyclohexanone and the hydrogenation of FUR, as well as the selectivity toward MF. By adjusting the Cu, Mn, and Si contents, a CuMnSi catalyst with the optimum stability, activity, and selectivity was prepared. Thus, for the catalyst with the optimum composition, at 279 °C and 0.1 MPa H_2_, a 94% yield to MF was achieved [153].

Even the direct conversion of xylose to MF was carried out, combining H*β* zeolite and Cu–ZnO–Al_2_O_3_ catalyst, in a continuous fixed-bed reactor, using *β*-butyrolactone/water as the solvent, achieving a yield of 86.8% for MF at 190 °C [87]. There was a cooperative effect between H*β* zeolite and *β*-butyrolactone that promoted the dehydration of xylose to FUR and enhanced the overall efficiency of the process. Moreover, the deactivated zeolite can be regenerated by calcination of carbon deposits, reaching similar activity as the fresh zeolite. These results showed that coupling process has advantages over the individual dehydrogenation and hydrogenation ones, such as avoiding the use of high H_2_ pressure, good energy efficiency, and environmentally benign process with an improved yield to MF.

On the other hand, sequential catalytic transfer hydrogenation/hydrogenolysis of FUR to MF was studied over in situ reduced Cu, Ni, and Pd/Fe_2_O_3_-supported catalysts, with 2-propanol as hydrogen donor, under batch and continuous flow conditions [87]. Cu and Ni/Fe_2_O_3_ catalysts yielded FOL with high selectivity (75 and 73%, respectively) at low conversion, but exhibited limited activity in the subsequent hydrogenolysis to MF. However, Pd/Fe_2_O_3_ showed an outstanding activity for MF formation, but also in the formation of ring-hydrogenation and decarbonylation products. The high activity of Pd/Fe_2_O_3_, in both transfer hydrogenation/hydrogenolysis, was attributed to a strong metal–support interaction. These results were in consonance with the preferential stabilization of the intermediate species by the different metal atoms, as previously stated [55,56]. Efficient magnetic Cu/FeO_x_ catalysts for CTH of FUR to produce MF with high yield (82.2%) were recently reported [155]. Small Cu nanoparticles fastened by FeO_x_ supporting layers were obtained under the appropriate calcination and reduction process. The small Cu clusters facilitated the hydrogenolysis of isopropanol to donate hydrogen, which favored the production of MF by dehydroxylation of rapidly formed FOL in the first reaction step. 

Some of these studies are based on the use of bimetallic Cu–noble metal catalysts, specifically bimetallic Cu–Pd ones. Chang et al. [101] studied CuPd/ZrO_2_ catalysts in the CTH of FUR in the presence of 2-propanol, as solvent and hydrogen donor. The selectivity to MF or MTHF could be modified by changing the Cu/Pd ratio, achieving a total yield of up to 83.9%. Mechanistic studies revealed that the main reaction pathways were firstly the hydrogenation of FUR to FOL and, then, the hydrogenolysis to MF and, simultaneously, the hydrogenation to MTHF. The catalysts showed high stability in reusing studies after five runs. Other bimetallic systems based on non-noble metals were also studied in this reaction. Thus, Fu et al. [156] studied the possible reaction pathways of conversion of FUR to MF over monometallic Cu and Ni, and bimetallic CuNi/Al_2_O_3_ catalysts, with formic acid as hydrogen donor. It was found that monometallic Cu catalysts showed a moderate catalytic activity, but a high selectivity toward the reduction of carbonyl to FOL, due to the repulsion of the C atom of adsorbed carbonyl C and surface metallic Cu atoms because of its oxophilic nature, which disfavored the decarbonylation process. On the contrary, monometallic Ni catalysts showed catalytic activity in both decarbonylation and hydrogenation, because the carbonyl C atom and carbonyl O atom were both simultaneously activated, which produces FOL and MF. The formation of furan was favored at high temperatures, through decarbonylation process, and ring-open reactions also happen due to the strong interaction of Ni metal sites with the furan ring. Bimetallic NiCu catalysts exhibited a synergistic effect of the two metals, due to a change of the adsorption configuration of FUR on the catalyst surface, with increased affinity for carbonyl and repulsion for furan ring, which promoted the formation of MF with high yield (92 mol%, at 210 °C, in isopropanol, after 7 h of reaction). Another efficient and recyclable bimetallic catalyst for furfural HDO was that based on CuRe supported on Al_2_O_3_, which allows attaining a 94% MF yield at 220 °C, after 4 h, by using isopropanol as the H-donor and solvent [157]. The synergy between Cu and Al_2_O_3_ enhanced the transfer hydrogenation of FUR, and Re species not only promoted the hydrogenolysis of FOL, but also the stability of the bimetallic catalyst to be recycled five times. 

A low-cost, selective, and reusable Cu–Co/γ-Al_2_O_3_ catalyst was used in the last part of a novel process designed to produce MF from biomass, in which the formation of a mixed Cu–Co phase seemed to be decisive for obtaining high MF yields [162]. The process includes the production of aqueous FUR from corncob biomass, the extraction of FUR from the aqueous phase, and its selective conversion into MF. The use of MTHF as solvent was the key factor in the global process, because it was used to extract FUR from the aqueous solution, provided high selectivity to MF from FUR (80%), and a complete MF and MTHF separation was not necessary after reaction, because MTHF presents suitable biofuel properties in the gasoline range. The use of another effective low-cost catalyst in this reaction was reported in a very recent work of Niu et al. [158]. A 72% MF yield, under ordinary pressure of N_2_ at 180 °C, in the CTH of FUR in isopropanol, was obtained over a family of low-cost CuZnAl catalysts. FOL was immediately produced, but for the cleavage of C–OH, the presence of Cu^0^ and Cu^+^ species on the surface was necessary with the preservation of spinel CuAl_2_O_4_ phase. The formation of these phases varied depending on chemical composition of catalysts, pretreatment, and reaction conditions. Thus, a synergistic effect was revealed for the trimetallic CuZnAl catalysts, since neither CuZn- nor ZnAl-based catalysts were effective for the cleavage of C–OH in FOL under the same experimental conditions. They concluded that Zn favored the formation of CuAl_2_O_4_ phase and Al facilitated the dispersion of Cu species on its surface, and both factors were important to obtain a high MF yield. Recently, Kalong et al. [109] studied the use of Ni- and Co-promoted Cu/*γ*-Al_2_O_3_ catalysts in the CTH of FUR, using 2-propanol as a hydrogen source. The production of MF was related to the amount of Lewis acid sites on the catalyst surface. Thus, when Co and Ni were incorporated into the Cu/*γ*-Al_2_O_3_ catalyst, MF yield increased in the order of Cu/*γ*-Al_2_O_3_ (20.7%) < CoCu/*γ*-Al_2_O_3_ (29.0%) < NiCu/*γ*-Al_2_O_3_ (41.1%), when the reaction was conducted at 200 °C for 2 h. The superior performance of Ni- and Co-promoted catalysts was due to the stronger Lewis acid sites on the catalytic surface, which could improve the ability of scission of C–OH bond via Lewis acid sites to generate MF. 

Another key factor in CTH processes is the stability of catalysts under the experimental conditions used. A novel study reported the selective transformation of FUR to MF with a high yield (90%) at 220 °C, without exogenous H_2_, in methanol. Li et al. [159] used a copper phyllosilicate precursor to obtain a highly water-resistant active Cu^0^–Cu_2_O/SiO_2_ catalyst for conversion of biomass in water at high temperature. These authors proposed a new catalytic process, which resulted in a better thermal balance, where endothermic methanol dehydrogenation and exothermic FUR hydrogenation were involved. Methanol was also used as H-donor in the H-transfer transformation of FUR, by using CuO and Cu_2_O [160]. In the proposed mechanism, adsorption and reforming of methanol firstly occurred and, then, a partial reduction of both Cu^2+^ and Cu^+^ to Cu^0^ took place. The presence of Cu^0^ species and Brønsted/Lewis acid sites associated with CuO_x_ were crucial for FUR HDO. The selectivity to FOL or MF highly depended on the hydrogen-production capability via methanol reforming and FUR adsorption strength over catalytic surface. Thus, CuO provided a higher MF yield (60%) than Cu_2_O due to the lower free energy and reaction energy barrier for methanol reforming and FUR transfer hydrogenation.

On the other hand, works about the metal oxide catalyzed transfer hydrogenation of FUR into MF are scarce. Recently, MFe_2_O_4_ (M = Cu^2+^, Ni^2+^ and Fe^2+^) inverse spinel catalysts were used in this reaction. FOL was the major product for NiFe_2_O_4_ and Fe_3_O_4_ catalysts; however, a 97.6% MF selectivity with a 99.4% FUR conversion was obtained by using CuFe_2_O_4_ at 200 °C after 1.5 h of reaction, with isopropanol as hydrogen donor [161]. It was concluded that Fe^3+^ ions present in CuFe_2_O_4_ catalyst were the active Lewis acid sites responsible for the greater interaction with the hydroxyl group of FOL, which produces the elongation of C–O and C–H bonds of the –CH_2_OH group and the production of MF in a higher selectivity. In addition, Cu^2+^ and Ni^2+^ ions showed a stronger interaction with isopropanol.

As shown in this section, although remarkable progress has been made in recent years in the production of MF from FUR by using copper-based catalysts, this is still a very interesting field of research because many of them have poor selectivity, high cost, or suffer deactivation. Therefore, it is currently a challenge to develop stable and effective copper-based catalysts to carry out this reaction with industrial prospects. To provide insights into the rational design of such catalysts, as has been shown in the studies reviewed in this section, the bifunctional nature of the catalysts is critical to obtain effective HDO of the furanic ring by combining the presence of Cu^0^ and Brønsted/Lewis acid sites.

### 2.4. Hydrogenation of Furfural to Cyclopentanone

The use of solid acid supports, such as ZrO_2_, is also necessary to attain a high activity towards the formation of cyclopentanone (CPO) from furfural [163]. In this sense, it has been reported that Cu^0^ sites are responsible for the dissociation of H_2_ and the hydrogenation of FUR to FOL. The presence of Cu^+^ species can also display an important role in the hydrogenation reaction, since Cu^+^ sites are electrophilic sites that can polarize the C=O bond of FUR through the isolated electron pair of carbonyl oxygen. This polarization can be increased by the incorporation of CoO_x_ species, since these Co^2+/3+^ species can also interact with the C=O group of FUR [164]. Then, Lewis acid sites, provided by ZrO_2_, can accept the electron of the carbonyl group, which favors the rearrangement of FOL, since ZrO_2_ stabilizes carbocations obtained as intermediate in the formation of 2-cyclopentenone. Finally, this compound is hydrogenated to form CPO as a final product [163]. Similar results were obtained for CuCo@C catalysts, where Cu and Co species interact strongly in such a way that the Co species exerts a promoter effect on the catalytic behavior of the Cu species. In this catalytic system, Lewis acid sites, required for the rearrangement of the FOL, are provided by the unreduced Cu^+^ and Co^2+^ species [165]. Another parameter that must be considered in the FUR hydrogenation in liquid phase is the effect of the solvent, since its polarity strongly affects the solvent–FUR interactions. Thus, the best catalytic activity was obtained with those solvents with low polarity. The low FUR conversion in solvents with high polarity is ascribed to the low solubility of FUR, although the use of polar solvents, such as H_2_O, can also facilitate the rearrangement of the FUR, leading to CPO. On the other hand, the use of apolar solvents, such as hexane or toluene, hardly favor catalytic activity due to the strong interaction between FUR and solvent molecules [164].

Cu-based catalysts, synthesized from Cu/Zn/Al hydrotalcite by Wang et al., are highly active towards cyclopentanol (CPOL) [166]. This compound is considered a valuable product, since it is widely used as solvent, as well as a starting compound for the synthesis of fragrances, fungicides, or drugs [2]. The calcination conditions of hydrotalcites seem to determine the catalytic behavior, since the dispersion of Cu species has an important impact on the catalyst morphology. Thus, small Cu nanoparticles improve the cyclopentanol yield. As was indicated previously, the coexistence of the metallic (Cu^0^) and Lewis acid sites (Al_2_O_3_) favors the formation of CPO; however, more severe experimental conditions also allow the reduction of CPO to CPOL [166].

## 3. Hydrogenation of Levulinic Acid to *γ*-Valerolactone

**Scheme 3:**
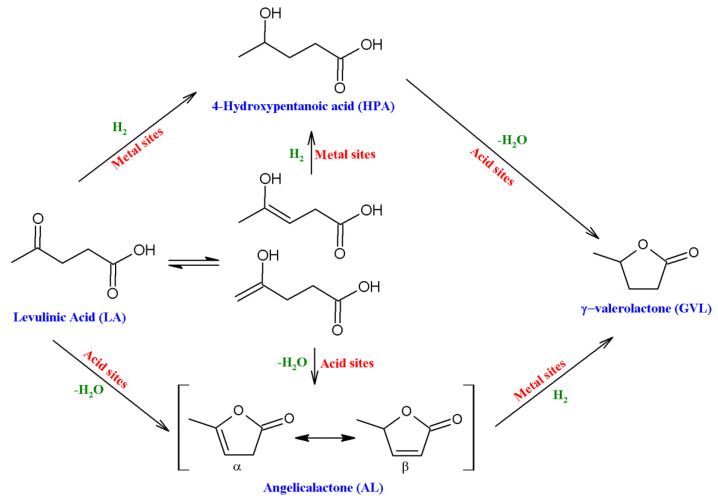
Different mechanistic pathways for LA hydrogenation to GVL.

γ-valerolactone (GVL) is considered, as previously stated, as a versatile building block derived from renewable feedstocks (lignocellulose) for the production of valuable chemicals and high-grade fuels, and it can be obtained by hydrogenation of levulinic acid (LA). Regarding the catalytic systems used to perform this hydrogenation process, Zhu et al., suggested the use of Cu-based catalysts, since these materials show higher activity towards C–O bond cleavage compared to C–C bonds [151]. Firstly, Christian Jr. et al., employed CuO–Cr_2_O_3_ for hydrogenation of LA, attaining a GVL yield of 62%. Later, Quaker Oats described a continuous process for the vapor-phase LA hydrogenation to GVL by using mixed-metal oxides CuO–Cr_2_O_3_ as catalysts [167]. Since then, the use of Cu-based catalysts for hydrogenation of LA in liquid and gas phases has already been reported. Two different pathways have been proposed for LA hydrogenation: i) dehydration to angelica lactone (AL) over acid sites and subsequent reduction to GVL over metal sites, or ii) reduction of LA to 4-hydroxypentanoic acid (HPA) over metal sites, followed by dehydration to form GVL (Scheme 3) [168,169]. Therefore, in the present section, the catalytic performance of most relevant Cu-based catalysts used for LA hydrogenation and its effect on the mechanism of this reaction will be summarized.

Thus, Hengne et al., studied several copper catalysts for LA hydrogenation, finding that Cu–ZrO_2_ and Cu–Al_2_O_3_ nanocomposites showed the best catalytic performance [11]. It should be noted that Cu in combination with Zr or Al oxides provided full LA conversion and total GVL selectivity (Table 4), while values of conversion between 4 and 45% were attained by using Cu with other metal oxides. They also stated that ZrO_2_ exhibited higher stability than other dopant metal oxides in aqueous solutions, under high temperature reaction conditions.

Jones et al., prepared a series of Cu–ZrO_2_ catalysts by co-precipitation for the hydrogenation of LA to GVL [171]. They observed that the Cu/Zr molar ratio had a strong influence on the BET surface area of catalysts, leading to higher activities for catalysts prepared with a Cu/Zr molar ratio of 1, attaining high GVL yields (y_GVL_ = 80%). Later, Ishikawa et al., prepared Cu–ZrO_2_ catalysts by two methods: methanothermal and oxalate gel precipitation [29]. The catalysts prepared by the first method were not active in LA hydrogenation, but the oxalate gel precipitation method led to very promising catalysts, with GVL yields close to 100%. Recently, this research group reported a novel pH gradient methodology to synthesize a series of Cu–ZrO_2_ catalysts for LA hydrogenation to GVL, demonstrating to be selective to GVL and more stable than those obtained by other co-precipitation methods [185]. They concluded that the activity of Cu–ZrO_2_ catalysts was dominated by the amount of Cu–Zr interface sites. On the other hand, they evaluated the influence of metal dopants on Cu–ZrO_2_ catalyst on the hydrogenation of LA [172], demonstrating that Sc, Cr, Fe, Co, and Zn had a detrimental effect on the catalytic performance, but the doping with Mn considerably enhanced GVL yield in such a way that the highest GVL yield (y_GVL_ = 82%) was attained with a Mn loading of 1 wt%. The catalytic activity of Mn/Cu–ZrO_2_ was 1.6 times higher than that found for the undoped Cu–ZrO_2_ catalyst, since the addition of manganese reduced the activation energy for LA hydrogenation. Moreover, they demonstrated, by ^13^C NMR and ^1^HNMR spectroscopies, that enolization of LA took place under the reaction conditions used, which had not been previously detected for typical Ru-based catalysts. Thus, Hirayama et al. [172] stated that the additional possibility of hydrogenation of the C=C bond of enol isomers to produce HPA should be considered (Scheme 3), since these enols possess a higher affinity for Cu surface than LA. 

**Figure 5:**
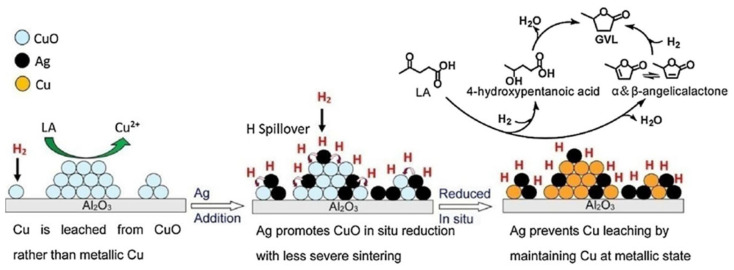
Schematic of the proposed mechanisms of in situ reduction and metal leaching prevention and the reaction pathway. Reprinted with permission from [175]. Copyright 2018 Elsevier.

Likewise, bimetallic catalysts are widely studied for hydrogenation reactions, since these materials tend to exhibit better catalytic performances for liquid-phase hydrogenation of LA to GVL. Thus, Obregón et al., evaluated bimetallic Ni–Cu supported over γ-Al_2_O_3_ for the hydrogenolysis of levulinic acid, attaining high GVL yield (y_GVL_ = 96%) after 2 h at 250 °C and 6.5 MPa H_2_ [173]. They observed that, although the catalytic activity decreased for Cu-based catalyst compared with Ni- and Ni–Cu-based catalysts, the presence of Cu inhibited the catalyst deactivation due to that lower α-angelica lactone (AL) yields were detected for Cu-based catalysts, since it is known that AL tends to polymerize in the presence of acids, resulting in selectivity losses and catalytic deactivation caused by carbon deposition [168]. They postulated that AL could come from the GVL previously formed, or AL desorbed from the catalyst surface. Later, these same authors corroborated the reversibility of the AL↔GVL reaction, carrying out the reaction from GVL and detecting significant amounts of α-angelica lactone [186]. Furthermore, Cu-based catalysts derived from hydrotalcites were also evaluated for LA hydrogenation. Hence, Yan et al., prepared Cu–Al, Cu–Cr, and Cu–Fe catalysts from their hydrotalcite precursors, which were highly selective for the hydrogenation of LA to GVL [124]. Although the best catalytic performance was attained with a Cu–Cr catalyst (y_GVL_ = 91%), high GVL yields were also obtained for Cu–Al and Cu–Fe catalysts (Table 4). They also detected pentanoic acid, which would be produced from dehydration of HPA, so these catalysts could follow this pathway to form GVL. Sunder et al., compared different Cu/Ni hydrotalcite-derived catalysts for liquid-phase hydrogenation of LA into GVL, by using dioxane as solvent [174]. They concluded that the Ni incorporation enhanced GVL selectivity, avoiding leaching of active Cu sites, due to the formation of a CuNi alloy, and the presence of Mg promoted the efficient LA hydrogenation into GVL, since the presence of surface Lewis basic sites activated the C=O group of LA. Recently, Zhang et al., studied the addition of Ag to a Cu-supported γ-Al_2_O_3_ catalyst to suppress Cu leaching in the hydrogenation of LA into GVL [175]. Thus, the incorporation of silver avoided the leaching and sintering of Cu species, since Ag facilitated the reduction in situ through hydrogen spillover and maintained that reduction state during the reaction (Figure 5). The CuAg/Al_2_O_3_ catalyst, without reduction pretreatment, provided complete LA conversion and almost total GVL selectivity at 180 °C by using tetrahydrofuran as solvent. No reaction intermediates were detected in the presence of CuAg/Al_2_O_3_ catalyst, probably due to that the formation of this intermediate was slower than their subsequent steps. In this case, Cu and Ag catalyzed hydrogenation, and γ-Al_2_O_3_ promoted the dehydration reaction.

On the other hand, the use of high H_2_ pressures involves high cost, complex reactor design, safety risk, and restriction in operation, so the catalytic transfer hydrogenation, as alternative hydrogenation route, is also being evaluated to replace H_2_ by using protic solvents, such as formic acid (FA) and 2-propanol [4,10]. Guo et al., synthesized Cu-functional carbon (Cu/CuO–FC) catalysts and evaluated their catalytic performance for LA hydrogenation into GVL in supercritical CO_2_–ionic liquid ((BMIM)Cl) systems, with FA as H-donor source [176]. They demonstrated that the use of supercritical CO_2_ considerably enhanced GVL yields, achieving 50% after 6 h at 170 °C. This supercritical CO_2_ exhibited a key role in LA hydrogenation, since it inhibited the volatilization of H-donor source, FA, increased mass transfer, and hydrogen solubility in the ionic liquid phase, and transported the GVL formed from the ionic liquid phase into the CO_2_ phase, avoiding its further hydrogenation to other chemicals, such as 2-methyltetrahydrofuran and 4-pentenoic acid.

Although it has been demonstrated that relevant GVL yields can be achieved in the presence of Cu-based catalysts for LA hydrogenation in liquid phase, the liquid conversion of LA to GVL requires high pressures and purification, in addition to generating waste product emissions; therefore, the gas-phase LA hydrogenation is considered to be an interesting alternative to produce GVL [183]. Bonrath et al., tested different Cu-based catalysts for gas-phase hydrogenation of LA to GVL and found that a copper-oxide-based one (50–75% CuO, 20–25% SiO_2_, 1–5% graphite, and 0.1–1% CuCO_3_/Cu(OH)_2_) provided a GVL yield of 93% for 4 days, without significant loss of catalytic activity or selectivity [112]. Putrakumar et al., studied Cu catalysts supported on γ-Al_2_O_3_ for gas-phase hydrogenation of LA to GVL, observing that the catalytic activity was directly related to the dispersion of Cu species and catalyst acidity [113]. Thus, they obtained GVL as a major product with a yield of 85%, but minor products such as angelica lactone and valeric acid were also detected. Therefore, this reaction proceeded through two consecutive steps: (1) LA dehydration to AL catalyzed by acid sites, mainly Lewis-type, of alumina; and (2) hydrogenation of AL to GVL on metal Cu sites (Scheme 3). This same research group corroborated that the catalytic activity and GVL selectivity were directly related to catalyst acidity and copper using Cu/ZrO_2_ catalysts for gas-phase hydrogenation of LA [177]. They also suggested that the increase in reaction temperature favors the conversion of LA by dehydration, but it provoked a decrease in GVL selectivity, increasing selectivities to AL and pentanoic acid. This latter is due to high temperatures provoking the ring-opening of GVL to produce pentanoic acid, which would be subsequently hydrogenated to pentanoic acid (valeric acid). In this study, they also compared Cu supported on different supports (ZrO_2_, Al_2_O_3_, SiO_2_, and TiO_2_) and concluded that catalytic activity was independent of the specific surface area, Cu–Al_2_O_3_ being the one that provided the highest LA conversion and GVL selectivity. On the other hand, Zhang et al., prepared Cu–ZnO catalysts supported on multiwalled carbon nanotubes by depositing Zn–hybrid film with controlled thickness by molecular layer deposition, and employed these catalysts for LA hydrogenation into GVL [178]. These catalysts reduced the activation energy, enhancing selectivity, efficiency, and stability due to Cu–ZnO interface sites (Cu^0^Zn) and the combined effect between Cu^0^Zn and Cu^+^ species. Thus, Cu–Zn catalysts synthesized by molecular layer deposition provided better catalytic performance than the catalyst without incorporation of Zn. They modified the amount of Cu^0^Zn sites, varying the cycle number of molecular layer deposition, and found a correlation between the ratio of Cu species (Cu^0^, Cu^+^, and Cu^0^Zn) and catalytic activity, in such a way that Cu^0^Zn is the key Cu species to induce high activity for LA hydrogenation. Sun et al., compared Cu-based catalysts over SiO_2_ and Al_2_O_3_ to Ni- and Co-based catalysts for gas-phase hydrogenation of LA [179]. In the case of Cu-based catalysts, Cu/Al_2_O_3_ exhibited better catalytic performance than Cu/SiO_2_ (Table 4). The presence of AL was not detected when Cu was supported over alumina, but it was observed for Cu/SiO_2_. The order of catalytic activity and stability was Ni/SiO_2_ > Cu/Al_2_O_3_ > Co/SiO_2_, while Cu/Al_2_O_3_ was the most selective. Although these authors suggested that GVL was mainly produced via HPA, the pathway via AL could not be discarded. Yoshida et al., prepared Cu–Ni/SiO_2_ catalysts, evaluating the effect of citric acid used for the catalyst synthesis to improve the dispersion [20]. They showed that the use of citric acid generated carbon residuals, which had an important role in stabilizing dispersed metal species on support and improved the catalytic activity. Thus, a Cu–Ni/SiO_2_ catalyst with 4 wt% Cu and 16 wt% Ni led to high values of LA conversion and GVL yield (C = 98% and y_GVL_ = 96%). Moreover, the addition of citric acid probably inhibited the Cu metal leaching. They also confirmed that the hydrogenation of LA to GVL took place from AL as intermediate.

On the other hand, Upare et al., evaluated the catalytic behavior of copper/silica nanocomposites in the gas-phase hydrogenation of LA [14]. They studied the influence of H_2_ pressure on LA hydrogenation, using a catalyst with 5 wt% Cu in dioxane, attaining total LA conversion and GVL selectivity of 94% at atmospheric pressure, which increased until 99.9% when the H_2_ pressure rose to 1 MPa. However, the GVL selectivity decreased to 93% when pressure was 2.5 MPa and led to the formation of 1,4-pentanediol. This catalyst also showed a high stability for more than 100 h, without deactivation or sintering of copper. Later, this research group evaluated the promoter effect of Ni on Cu/SiO_2_ nanocomposites in LA hydrogenation, using FA as hydrogen source [180]. Firstly, without Ni, an LA conversion of 66% and selectivity of 45% GVL and 55% AL were attained. However, the presence of Ni enhanced both LA conversion and GVL selectivity, achieving a maximum conversion of 98% and GVL selectivity of 92% by using a Ni loading of 20 wt% after 100 h of time-on-stream at 265 °C. The addition of Ni also improved the thermal stability of Cu/SiO_2_ nanocomposite catalysts, preventing the Cu sintering after long reaction time (200 h). Yoshida et al., also studied bimetallic Cu–Ni over SiO_2_ catalysts in gas-phase hydrogenation of LA to GVL in the presence of H_2,_ reaching a GVL yield of 99% after 50 h at 250 °C [181]. Although GVL was the major product, other byproducts, such as AL, acetone, and methyl ethyl ketone (MEK), were detected under different reaction conditions. It is known that AL is produced by the cyclodehydration of LA, and MEK and acetone are products derived by LA decomposition. The presence of Ni improved the hydrogenation ability of Cu, but it generated decomposition products, since Cu was inactive for the C–C cleavage of LA. Therefore, bimetallic Cu–Ni catalysts enhanced the catalytic performance compared to their pertinent monometallic ones due to the fact that Ni improved the hydrogenation ability and reduced the carbon accumulation, and Cu inhibited the LA decomposition.

Likewise, Cu–SiO_2_ catalysts over different silica supports were tested for LA hydrogenation to GVL by Lomate et al., using formic acid as hydrogen source [182]. They detected GVL as a major product, and angelica lactone and pentanoic acid as minor products. These authors checked that hydrogenation did not take place when support without copper alone was employed as catalyst, with AL being the main product obtained through an acid catalyzed reaction. With regard to Cu–SiO_2_ catalysts, they concluded that the presence of higher amount of single Cu^2+^ species and Lewis acid sites with medium acid strength was relevant for LA conversion to GVL, with the crystallite size being less important. They also speculated that Lewis acid sites are more important than Brønsted-type ones for LA hydrogenation into GVL. Moreover, the presence of strong acid sites combined with high reaction temperature could favor the ring-opening of GVL to give pentanoic acid. In addition, the effect of FA concentration was studied by varying the LA:FA ratio. Due to the fact that the selectivity of angelica lactone depended on FA concentration and, consequently, on GVL selectivity, these authors concluded that the reaction first proceeded by dehydration to AL, followed by hydrogenation to GVL, although the formation of GVL from hydroxypentanoic acid could not be ruled out. Later, these authors corroborated this conclusion, since they detected AL as intermediate, indicating that the hydrogenation of AL in the presence of FA and water occurred via the LA–AL–GVL route [183]. They also compared Cu-based catalysts over supports with different acid–basic properties (Al_2_O_3_, SiO_2_, TiO_2_, ZSM-5, and SiO_2_–Al_2_O_3_), with Cu/SiO_2_ being the catalyst that showed higher values of LA conversion and GVL selectivity. In the case of Cu/TiO_2_, the lowest LA conversion and GVL selectivity were found, but it had the highest selectivity to pentanoic acid. In all cases, the presence of AL was detected, confirming that LA hydrogenation took place via AL. Indeed, higher values of AL selectivity were observed for Cu–ZSM-5, Cu–SAL, and Cu–TiO_2_ due to the presence of higher amount of acid sites, which favor the dehydration process. However, the Cu sites in these catalysts were less active to convert AL to GVL. Due to the fact that a clear relationship between acidity and catalytic performance could not be established, they concluded that the nature and amount of acid sites is not a key determining factor in the LA hydrogenation to GVL, in spite of the fact that the presence of acid sites is required by dehydration. Recently, Ashokraju et al., prepared Cu/Fe_2_O_3_ catalysts using mesoporous SBA-15 silica as support for gas-phase hydrogenation of LA using FA [184]. They pointed out that Fe_2_O_3_ could catalyze both formic acid decomposition and levulinic acid hydrogenation, giving rise to GVL with LA conversion and GVL selectivity of 70 and 81%, respectively. After Cu incorporation, full LA conversion with 100% selectivity toward GVL was attained. They related this excellent catalytic performance to the interaction between Cu and Fe_2_O_3_, in such a way that a larger amount of easily reducible CuO species was present. However, a slight deactivation was detected after 8 h of time-on-stream, which was attributed to the formation of coke on the catalyst surface.

Therefore, it should be taken into account that angelica lactone has been found more often as an intermediate for hydrogenation of levulinic acid than 4-hydroxypentanoic acid, so it seems that this pathway is more likely when bifunctional acid/metal catalysts are employed. However, the presence of 4-hydroxypentanoic acid as reaction intermediate in the GVL production cannot be discarded due to the fact that this product is more unstable [182]. Considering that bifunctional catalysts are required for this process, it would be of great interest to confirm if these materials are able, or not, to convert directly lignocellulosic biomass in GVL. The presence of both acid and metal sites could lead to hydrolysis of different biomass fractions and subsequent dehydration and hydrogenation reactions. Therefore, these Cu-based catalysts should be tested by using lignocellulosic biomass as feedstock.

### 3.1. Hydrogenation of Alkyl Levulinate to γ-Valerolactone

**Scheme 4:**
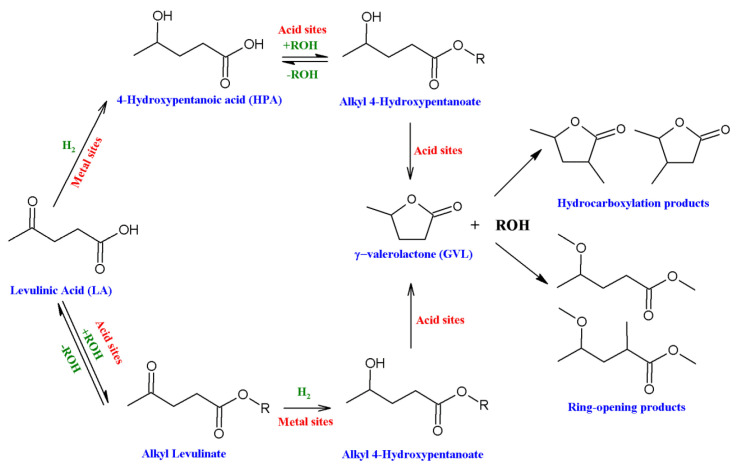
Different mechanistic pathways for LA hydrogenation to GVL in presence of an alcohol.

Although the GVL production from levulinic acid has been largely reported in the literature, the use of alkyl levulinates, which can be directly produced from carbohydrates, is currently considered as an interesting alternative to obtain GVL, due to their features of nonacidity and easy separation [187]. Moreover, the formation of humins can be suppressed in alcoholic medium [188,189]. Therefore, the LA hydrogenation in presence of an alcohol can occur through two possible pathways: (i) the hydrogenation of LA into HPA followed by esterification with alcohol and subsequent acid-catalyzed conversion to GVL; and (ii) the esterification of LA with alcohol followed by hydrogenation and subsequent acid-catalyzed transformation to GVL (Scheme 4) [190].

Thus, the use of different alcohols as solvents for LA hydrogenation has been reported in the literature. Hengne et al., who studied nanocomposites of Cu–ZrO_2_ and Cu–Al_2_O_3_ for LA hydrogenation, also carried out the hydrogenation of LA in methanol [11]. Unlike the results that they found by using water as solvent, the GVL selectivity decreased for different metals than Zr and Al, such as Cr or Ba, in the presence of methanol, due to their poor acidity, with a lower esterification rate. In the case of Cu–ZrO_2_ catalysts, full conversion and 90% GVL yield were achieved (Table 5). However, a slight decrease in GVL selectivity was observed, which can be due to less facile cyclization of methyl 4-hydroxypentanoate having a bulkier methyl group, compared to that of 4-hydroxypentanoic acid. It should be noted that both Cu–ZrO_2_ and Cu–Al_2_O_3_ provided complete formation of methyl levulinate (ML), after 30 min of reaction, and the hydrogenation of methyl levulinate did not begin until the transformation of LA to ML was complete, indicating that the adsorption of LA and ML are competitive. They concluded that the LA hydrogenation in methanol took place via esterification and subsequent hydrogenation to GVL. Later, Zhang et al., reported that a higher GVL yield can be attained if some water is added to methanol, compared to the use of pure methanol as solvent, using a magnetic Ni/Cu/Al/Fe catalyst, because the GVL formation is faster from LA as feed [11]. As methyl 4-hydroxypentanoate was not detected, these authors suggested that the GVL production could happen via hydrogenation of LA into HPA, followed by esterification with methanol and subsequent acid catalyzed into GVL (1) or via esterification of LA with methanol into ML followed by direct hydrogenation into GVL (2), without transformation to methyl 4-hydroxypentanoate, which is not in agreement with that previously reported in the literature [190]. They concluded that the reaction was processed via pathway 2 under reaction conditions employed, since the route for GVL production depended on the reaction temperature. Sun et al., directly carried out the gas-phase hydrogenation of ML to GVL and compared Cu-, Ni-, and Co-supported over SiO_2_ or Al_2_O_3_ catalysts [179]. They found that Cu–Al_2_O_3_ showed the highest GVL selectivity from ML, but the conversion decreased with time-on-stream due to coke formed on the catalyst surface. They also proposed methyl 4-hydroxypentanoate as a possible reaction intermediate, in spite of the fact that this product was not detected, because it is easily converted to GVL through lactonization.

On the other hand, ethanol has also been studied as solvent for LA hydrogenation for GVL production. Xu et al., tested several supported Cu catalysts, but the major product was ethyl levulinate (EL) in most of the cases, and hydrogenation was barely detected by using ZSM-5, ZrO_2_, SiO_2_, and Ta_2_O_5_ as supports [23]. Only Cu supported on γAl_2_O_3_ and Cu–WO_3_/ZrO_2_ prepared by co-precipitation provided high GVL yield (y_GVL_ = 93 and 94%, respectively) (Table 5). They found that the incorporation of W improved the GVL selectivity due to the interaction between Cu and W, in such a way that GVL yield rose with the increase in W loading, being maximum for 10 wt%. Likewise, they observed that GVL was enhanced at higher reaction temperature, with maximum at 200 °C, since a further increase in temperature favored overhydrogenation products such as pentanoic acid or ethyl pentanoate. Zheng et al., prepared Cu/SiO_2_ and Al_2_O_3_-doped Cu/SiO_2_ catalysts by co-precipitation and evaluated their catalytic performance in hydrogenation of ethyl levulinate to GVL [194]. The doping with alumina considerably improved the stability of catalyst, which provided full EL conversion, and the selectivity to GVL + 2-methyltetrahydrofuran (MTHF) was higher than 94% after 100 h of reaction. This fact may be due to the fact that doping with alumina reduced the particle size of CuO and sintering of Cu particles was negligible, consequently, improving stability. They also reported that the relative ratio of GVL and MTHF could be adjusted by varying the reaction temperature, in such a way that GVL is the major product below 240 °C and MTHF selectivity enhances increasing the temperature to 250 °C. They also proposed that EL is hydrogenated to form ethyl 4-hydroxypentanoate, whose cyclization led to GVL and ethanol production. MTHF could also be obtained due to the further hydrogenation of GVL. However, the use of bifunctional catalysts with acid and metal sites could provoke the formation of ethyl pentanoate due to the catalyst acidity.

Recently, the use of alcohols as solvent and H-donor for the transfer hydrogenation of levulinate esters through Meerwein–Ponndorf–Verley (MPV) reduction has received considerable attention [21,195,196]. Considering that methanol is the cheapest alcohol compared to other aliphatic ones, its use as H-donor would be advisable. Although methanol possesses the worst dehydrogenation capacity in MPV reduction, it can be catalytically reformed to H_2_ and CO_2_/CO, being a promising hydrogen source [193,197]. Thus, methanol can act as hydrogen source for hydrogenation reaction and participate in the formation of methyl levulinate (ML) in the presence of acid sites. Therefore, a bifunctional catalyst able to carry out reforming of methanol and hydrogenation of methyl levulinate in methanol is required as solvent, being less corrosive than if FA is employed [21]. Hence, Tang et al., carried out the hydrocyclization of methyl levulinate (ML) to GVL using a nanocopper catalyst without external source of H_2_. This nanocopper catalyst was reduced in situ by H_2_ produced by methanol reforming, and this catalyst possesses a dual role: H_2_ production by reforming of methanol, and the subsequent hydrogenation of ML to GVL. Thus, almost full ML conversion and GVL yield of 85% were attained after 1 h at 240 °C under N_2_ atmosphere. These authors affirmed that Cu-based catalysts could also promote the ring-opening of GVL in alcohol under high H_2_ pressures and, even hydrocarboxylation/alkoxycarbonylation products were detected as byproducts (Scheme 4). Nevertheless, they concluded that the hydrogenation of ML to methyl 4-hydroxypentanoate was the rate-limiting step, since this latter was not detected, with the ring-closing of this product to GVL being very fast for temperatures above 150 °C. It should be pointed out that this nanocopper catalyst is employed as a multifunctional catalyst, and competitive adsorption between methanol, ML, GVL, H_2_, CO, and CO_2_ succeed on the catalyst surface. This research group attained a GVL yield of 90% using self-supplied H_2_ coming from the decomposition of methanol in the presence of Cu–Cr oxide catalyst [191]. A considerable amount of GVL-derived products, such as pentanoates, and hydrocarboxylation ones were observed. Cao et al., synthesized Cu–MgO as a bifunctional catalyst for both H_2_ production by methanol reforming and hydrogenation of ML to GVL by using methanol as solvent [192]. Thus, a GVL yield of 91% was reached after 4 h at 220 °C without external H_2_ source, and the catalyst could be reused for at least seven catalytic runs (Table 5). Authors checked that these side reactions were favored at higher reaction temperatures due to the fact that Cu species were completely reduced, concluding that Cu^+^ species are conducive to suppressing the side reaction.

As was previously mentioned, methanol can give rise to H_2_ and CO/CO_2_ and subsequent hydrocyclization of ML to GVL, through methyl 4-hydroxypentanoate as intermediate, but this latter was not detected, confirming again that the hydrogenation of ML to methyl 4-hydroxypentanoate was the rate-limiting step. In addition, these authors corroborated that hydrocarboxylation/alkoxycarbonylation products could be formed due to the reaction between GVL and CO_2_ in methanol. They also affirmed that the presence of Cu^+^ species in Cu–MgO catalyst favored the ML hydrogenation due to the adsorption of ML preferentially taking place over these Cu^+^ species, because they are more electropositive than metallic Cu. Thus, the presence of MgO stabilized these Cu^+^ species, avoiding their reduction under H_2_ atmosphere, and promoting the ML hydrogenation to GVL. In the same way, CuO_x_–CaCO_3_ was demonstrated to be an efficient bifunctional catalyst for the hydrogenation of ML into GVL, by using methanol as hydrogen source, since this catalyst possesses Cu^+^ species that promote this hydrogenation process [193]. The increase in reaction temperature caused a decrease in GVL selectivity, due to that the side reactions have higher activation energies than ML hydrogenation, and the elevation of temperature until 280 °C resulted in complete reduction of copper species. A maximum GVL yield was achieved (y_GVL_ = 96%) after 3 h at 240 °C. In this work, hydrocarboxylation and ring-opening of GVL were also detected. 

Finally, the use of other alcohols able to donate hydrogen for the transfer hydrogenation of LA has generated great interest in the literature. Gong et al., employed 2-propanol as hydrogen donor and Cu supported over activated carbon as catalyst, attaining a GVL yield of about 90% [10]. The main intermediate detected was isopropyl levulinate obtained from the esterification of LA with 2-propanol. They proposed that AL firstly reacted with 2-propanol to form isopropyl levulinate followed by its conversion to GVL. A synergistic effect was found between Cu^0^ and Cu^+^ species, in such a way that electrophilic Cu^+^ species acted as Lewis acid sites to adsorb LA molecules and Cu^0^ species led to dehydrogenation of 2-propanol to generate active hydrogen. Moreover, they confirmed that LA conversion to GVL in alcohol took place through the formation of LA esters, followed by their hydrogenation to GVL.

Therefore, the GVL production from hydrogenation of LA, or its esters, in alcohol medium is considered of great interest, as inferred from the number of papers published on this topic. Thus, these alcohols act as solvent and hydrogen source, avoiding the use of high pressure of H_2_. In the case of methanol, it should be noted that the presence of CO and CO_2_, coming from methanol reforming, favors the formation of hydrocarboxylation/alkoxycarbonylation byproducts, decreasing GVL yield.

### 3.2. Hydrogenation of Levulinic Acid to 2-Methyltetrahydrofuran

**Scheme 5:**
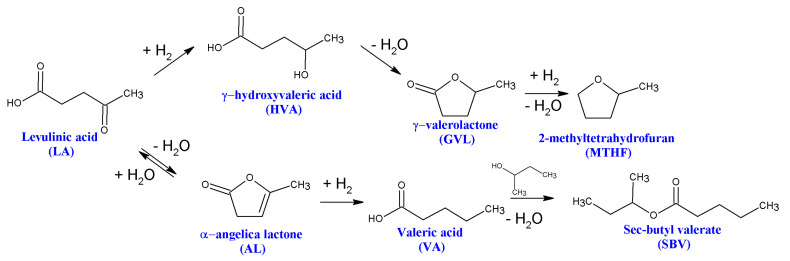
Catalytic routes of the hydrogenation of levulinic acid to 2-methyltetrahydrofuran.

Regarding the synthesis of MTHF, some processes have been designed by starting from different compounds. Thus, Dong et al., used furfural as the key intermediate [198], and Du et al., proposed GVL as such starting point for the MTHF synthesis [199], whereas some methods start from LA [14]. The direct and selective transformation of LA to 2-MTHF is a challenging process that requires harsh conditions. MTHF is typically produced via the sequential catalytic dehydrogenation-hydrogenation of LA (Scheme 5).

Different copper-based catalysts have been tested in the synthesis of MTFH from LA. Upare et al., reported a highly selective composite Cu/SiO_2_ catalyst prepared by a precipitation–deposition method [14]. The performance of composites was evaluated in the hydrogenation of LA (10 wt%) dissolved in 1,4-dioxane, in a stainless-steel fixed-bed reactor at 265 °C and moderate H_2_ pressures (1–5 MPa). First, the H_2_ pressure influence on the catalytic performance of catalysts with low copper loading was studied. At 265 °C and 0.1 MPa, Cu(5)/SiO_2_ showed 100% conversion of LA with selectivities to GVL of 94% and to *β*-angelica lactone (AL) of 6%, not detecting MTFH. As the H_2_ pressure increased to 1 MPa, the selectivity towards GVL increased to 99.9%. Further increases in pressure led to the formation of 1,4-pentanediol (PDO) with decreasing selectivity towards GVL (93%). The effect of the copper loading (5–80 wt% Cu) on the LA hydrogenation was also evaluated. At H_2_ 2.5 MPa and 265 °C, as the copper loading was increased, a great change of the selectivity pattern was observed, shifting the product selectivity from GVL to MTHF and 1-pentanol (PO). Thus, the yield of MTHF increased to 64% with an increase in the copper loading up to 80 wt%. When the effect of H_2_ pressure (1–5 MPa) was examined over Cu(80)/SiO_2_, the LA conversion reached 100% and the selectivities towards MTHF and PO were similar in the range 2.5–5 MPa, being 65 and 35%, respectively. From those results, it was inferred that lower copper loading favored the selective hydrocyclization of LA to GVL, while higher copper loading led a step forward to the hydrogenation to MTHF and 1-pentanol via GVL, as shown in Scheme 5. It was noticeable that the nanocomposite Cu/SiO_2_ catalysts with 5 and 80 wt% Cu exhibited stable activities for more than 100 h, without deactivation or important copper sintering. This plausible mechanism was confirmed by the hydrogenation of GVL over Cu(80)/SiO_2_, yielding similar MTFH and PO selectivities to when LA was used. This means that the sequential reaction of LA to MTHF via GVL is the main reaction route. Finally, in order to improve the selectivity towards MTHF, the promoter influence of Ni was studied for the Cu(80)/SiO_2_ catalyst. The catalyst with 7 wt% of Ni (Ni–Cu(72)/SiO_2_) showed a very high selectivity towards MTHF (89%) without significant loss of catalytic activity for 320 h, indicating the promoting effect of Ni on the selectivity.

The influence of the reaction method on the catalytic activity of Cu supported on SiO_2_ MINT catalysts (MINT stands for microwave-induced nanotubes) was examined by Bermúdez et al. [200]. Two methods were selected: microwave-assisted batch reactor and flow reactor. In both methods, formic acid (FA) was selected as H_2_ source, the temperature was fixed at 150 °C, the reaction time was set at 30 min for the microwave-assisted reaction, and a contact time of 1 min was set for the flow reaction. The Cu–MINT catalysts were compared with a commercial Pd(5 wt%)/C catalyst. The microwave-assisted reaction was effective in decomposing FA in 30 min. The catalyst with 0.51 wt% Cu loading provided almost quantitative conversion of LA after a 30 min reaction, and the main products were the hydrogenated compounds, 1,4-pentanodiol (PDO) and MTHF, with selectivity values of 75 and 25%, respectively. The reutilization of the catalysts maintained over 90% of their initial activity after three reuses, and selectivities did not significantly change upon reuse, as well. The catalytic activity was ascribed to a combination of Cu^+^ and, most importantly, Cu^0^ species in conjunction with the MINT nanostructure. Interestingly, the Cu^+^ and Cu^0^ species were formed during the reaction via in situ reduction of CuO by the evolved H_2_ from FA decomposition. The best conditions found with the microwave-assisted reaction were translated to the flow reactor. Flow reactors offer important advantages such as control of reaction parameters, quick and efficient reagent mixing, and shorter times of reaction, as well as enhanced heat and mass transfer. The activities of Cu–MINT and commercial Pd(5 wt%)/C were found to be very similar under the investigated flow conditions (Cu–MINT 79 vs. 73% Pd/C), but remarkably different selectivities to MTHF were observed in both systems. Only two main products (MTHF and PDO) were obtained, with 60 and 40% selectivity, respectively, under the investigated conditions for Cu–MINT. On the other hand, the Pd(5 wt%)/C system gave rise to a number of hydrogenation products, including PDO, pentanoic acid (PA), and 4-hydroxyvaleric acid (HVA), with a low selectivity to MTHF (<30 mol%). Under flow conditions, the Cu–MINT catalyst was not stable, as it was significantly deactivated after 20 min of time-on-stream. This deactivation was attributed to the important amount of Cu leached in solution (62 ppm). From the above results, the authors proposed a mechanism for Cu-based materials, in which PDO is believed to be the key intermediate in the production of MTHF. This compound seemed to be produced via a two-step reaction from LA, first to *β*-angelica lactone (AL), whose subsequent hydrogenation gives rise to PDO (Scheme 5). PDO can be then readily converted into MTHF via dehydration under heating, and/or promoted by FA, due to the thermal instability of the compound.

Zheng et al., synthesized an Al_2_O_3_-doped Cu/SiO_2_ catalyst for the production of GVL and MTFH from ethyl levulinate (EL) [194]. The catalyst was prepared by the co-precipitation method and tested in a flow reactor. The EL (EL: ethanol volume ratio of 1:1) was injected in the reactor at WHSV of 0.6 h^−1^, the temperature ranged between 141 and 251 °C, and H_2_ pressure was varied between 1 and 3 MPa. Below 200 °C, EL was not completely converted, but at 230 °C, in the first 100 h, EL was almost entirely converted. However, the undoped Cu/SiO_2_ catalyst was drastically deactivated after 100 h of time-on-stream (TOS). The total selectivity toward GVL + 2-MTHF remained at around 90%, but the authors do not inform about the individual selectivity. The products were analyzed after 54 h TOS, with GVL being the main product, with selectivities towards 2-MTHF, GVL, 2-pentanol, 1-pentanol, 1,4-pentanediol, and ethyl valerate of 7.2, 85.1, 3, 1.2, 2.0, and 0.1%, respectively, in addition to 1.4% unidentified products. Therefore, the Cu/SiO_2_ catalyst is more selective to GVL than MTFH. The Cu/Al_2_O_3_–SiO_2_ was superior to that of Cu/SiO_2._ The conversion of EL on Cu/Al_2_O_3_–SiO_2_ was almost 100%, and the total selectivity of GVL + 2-MTHF was greater than 94% in 1000 h long-term test, indicating the robustness of this catalyst. The reaction temperature affected the MTFH and GVL selectivities; thus, at 141 °C, the MTFH selectivity was nil and the GVL selectivity was 96%, with 4% of unidentified compounds, but when the temperature was raised, the MTFH increased up to 65% at 250 °C, with a decrease in GVL selectivity until 8%. It is noticeable that the selectivity to the other compounds was largely suppressed, except 1-pentanol selectivity, which was 16%. The authors claimed that this catalyst was not convenient if MTFH was the target compound. From the detected compounds, the authors proposed a reaction pathway (Scheme 5). In a first step, EL was selectively saturated to form ethyl 4-hydroxypentanoate, whose cyclization leads to the formation of GVL and ethanol. The subsequent hydrogenation of GVL results in the formation of 2-MTHF under harsher conditions. Therefore, the sequential reaction of EL to 2-MTHF, via GVL, is the main reaction route.

**Figure 6:**
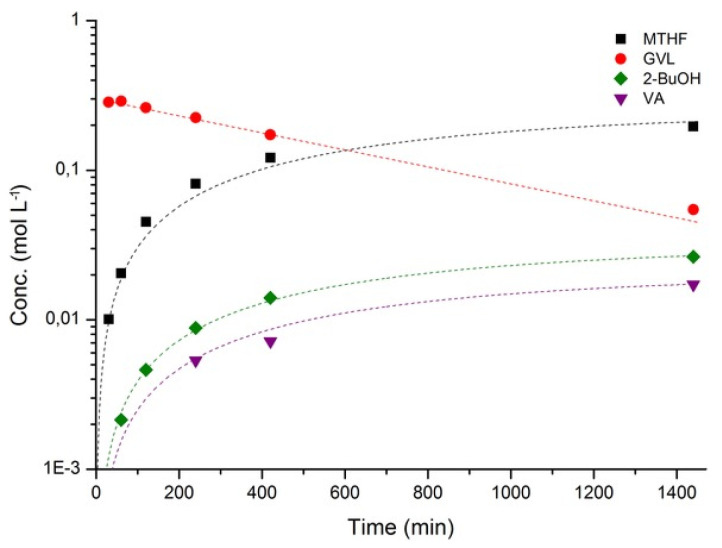
Concentration of the reaction products as function of the reaction time (dots) for 23Ni–12Cu/Al_2_O_3_ and data fitting (lines). Reaction conditions: 5 wt% LA in 2-PrOH, LA/cat. ratio 10 g/g, 230 °C reaction T, 70 bar H_2_. Reprinted with permission from [186]. Copyright 2015 Wiley and Sons.

Bimetallic Ni–Cu catalysts also proved to be active in the synthesis of MTHF from LA [201]. Obregón et al. [186] studied the hydrogenation of LA to MTHF over Ni–Cu/Al_2_O_3_ catalysts using green solvents, as water and alcohols derived from biomass, in a batch reactor and 7 MPa of H_2_ pressure. As water is the greenest solvent, Ni(35 wt%)/Al_2_O_3_ was tested with water solutions of LA, obtaining high GVL yields, but it was ineffective in producing MTHF, so other solvents (ethanol, 2-propanol and 2-butanol) were tested, reaching the best improvement when the reaction was carried out in 2-propanol (MTHF yield of 45.9%). This result was explained due to the excellent hydrogen-donor properties of such alcohol. The authors claimed that the production of H_2_ on the active sites of the catalyst dramatically increased the hydrogen availability and, hence, the GVL ring-opening reaction rate. This was also confirmed by the large amount of acetone detected along with the reaction products. The monometallic Cu(35 wt%) catalyst was also active for MTHF synthesis, reaching a yield of 22.7% when it was tested under similar experimental conditions. When the bimetallic catalysts were examined, the authors found a maximum 56% MTHF yield for a 23:12 Ni/Cu ratio. They also observed that the catalyst with higher Ni contents resulted in both lower MTHF yield, with a plateau around 44%, and lower GVL yield, although the monometallic Ni catalyst converted almost all GVL, but increased the formation of byproducts up to 24%. Therefore, high Cu loadings seemed to prevent side reactions, leading to byproducts such as 2-butanol, 1- and 2-pentanol, and valeric acid, and hence improved the selectivity towards MTHF, whereas Ni provided high activity in converting GVL (Scheme 5). This hypothesis was confirmed by a long-term reaction carried out with the monometallic Cu(35 wt%)/Al_2_O_3_, reaching an outstanding 75% MTHF yield (Figure 6). From XRD, H_2_-TPR, and XPS studies, the authors confirmed the formation of a Ni–Cu phase necessary to promote the conversion of the stable GVL and the selectivity towards MTHF. The stability of the 23Ni–12Cu/Al_2_O_3_ catalyst in the reaction media was also evaluated under at 250 °C and 5 h. When water was used as solvent, up to 2% of Ni was leached, but it was negligible using 2-propanol, corroborating both the promotional effect of 2-propanol in the yield of MTHF and the beneficial effect in stabilizing the catalyst particles.

On the other hand, Xie et al. [201] synthesized a family of mesoporous Al_2_O_3_–ZrO_2_ acidic supports with different Al/Zr molar ratios by the sol–gel method, and they were used as supports of Cu–Ni bimetallic catalysts. The authors studied the influence of the support composition, active metal, and reaction temperature and pressure on the LA hydrogenation, in an autoclave reactor and using 2-butanol as solvent. The influence on the catalytic performance of the acidic and textural properties of the supports was evaluated for a Cu and Ni loading of 10 wt%, respectively. It was observed that MTHF selectivity increased when the Al:Zr molar ratio increased from 1:0 (11.9%) to 9:1 (99.8%), at full conversion of LA. Although a similar synthesis method was used for the supports, the mixed-metal oxide had a higher concentration of strongly or weakly acidic sites and a higher total acidity than ZrO_2_, which favored the ring-opening/cyclodehydration of GVL into MTHF. Cu–Ni/Al_2_O_3_ gave a lower yield of MTHF (91.1%), which was ascribed to the lower BET surface area and surface acidity of the alumina support. These results indicate that both the porous structure and high surface acidity contributed to the excellent catalytic performance of Cu–Ni/Al–Zr(9). The Al–Zr(9) was selected as support to study the influence of the metallic loading of Cu and Ni. The monometallic Ni catalyst produced GVL (86.7% selectivity) and MTHF (1.6%), whereas the Cu-based catalyst yielded both MTHF (45.2%) and GVL (54.8%). For the bimetallic catalysts, the catalyst with 10 wt% Cu and 10 wt% Ni provided the highest MTHF selectivity, with a value of 99.8%. When the Cu loading was lowered, the selectivity to GVL was increased, indicating the low catalytic activity of Ni in GVL hydrogenation. On the contrary, when Cu loading was increased, a lower selectivity to MTHF was achieved, which can be explained by the lower hydrogenolysis activity of GVL. Moreover, the physical mixture of monometallic Cu/Al–Zr(9) and Ni/Al–Zr(9) catalysts gave poor MTHF selectivity (1.6%), indicating that the synergistic effect between Cu and Ni in the bimetallic Cu–Ni/Al–Zr(9) catalyst played a crucial role in the high selectivity. The study of the influence of reaction temperature and H_2_ pressure was evaluated on the Ni–Cu/Al–Zr(9) catalyst, showing that temperatures lower than 220 °C favored the production of GVL, whereas temperatures higher than 220 °C decreased the MTHF selectivity, owing to the generation of byproducts. In addition, the influence of H_2_ pressure at 220 °C resulted in a high yield of GVL at low H_2_ pressures, and when H_2_ pressure was increased, the MTHF selectivity was favored at the expense of GVL selectivity. The selectivity to MTHF reached 99.8% when H_2_ pressure exceeded 3 MPa. The evolution of reaction products with the time-on-stream showed that the LA conversion was 100% over Cu–Ni/Al–Zr(9) catalyst after 1 h and the yield of GVL decreased from 96.6 to 0% as the reaction proceeded, suggesting that GVL was the intermediate. MTHF yield increased continuously with the reaction time, and the maximum yield (99.8%) was reached after 10 h. The authors proposed a reaction pathway on the basis of the catalytic results (Scheme 5). The recyclability of Cu–Ni/Al–Zr (9) catalyst was also investigated, being reused for five catalytic cycles without a considerable loss of catalytic activity.

## 4. Future Outlooks

In this review, the importance of certain molecules derived from the hydrogenation of FUR was presented; among them, FOL, MF, and GVL. It was shown that FUR can be transformed into all of them using copper species as active phase. The Cu-based catalysts exhibited high values of activity and selectivity, as was described in this work. In addition, copper catalysts can be used in the CTH reaction, which presents a versatility that allows avoiding the use of molecular hydrogen as reducing agent, since hydrogen involves the use of high pressures, higher cost, and safety risk in operation. Finally, all these processes must be efficient, selective, and economical, reducing the negative impact on the environment.

Although there is extensive scientific literature based on the use of copper catalysts, there are currently some gaps that need to be addressed; among them are the following.

Nowadays, the role of the alcohols in the CTH over metals is not very clear. Most studies carried out use primary and, mainly, secondary alcohol, which are generally used as hydride donors. However, many studies are performed in H_2_ medium, so it is not clear what the role is of each hydrogenating source. In some cases, H_2_ cannot be activated by the presence of acid–base catalysts in such a way that the alcohol should be the unique H source to promote the reduction for FUR. This hydrogen donation leads to the dehydrogenation of aldehyde and ketones. However, these dehydrogenation products are generally unstable, forming acetals due to the acetalization of the aldehyde/ketone and alcohol under strong conditions. Moreover, the solvent-derived dehydrogenation products can also react with FUR via aldol condensation.

The analysis of kinetics studies to compare the intrinsic activity of the catalysts has not been carried out in depth yet. This comparison is very complex due to the fact that catalysts systems are very heterogeneous and the turnover number (TON) and turnover frequency (TOF) are not indicated in many studies. Different parameters, such as particle size, metal dispersion, support, and the catalyst stability, are demonstrated to have influence on the catalytic performance. Regarding the synthesis of the catalysts, a challenge is the design of single-atom or single-site catalysts to determine the catalytic behavior in the FUR hydrogenation per active site. Nowadays, these single-atom-based catalysts display low metal content, so the challenge is the synthesis of Cu-based catalyst with higher loading.

One of the strategies for increasing the activity or the stability of the copper catalysts was the presence of a second metal that contributes to the activation of hydrogen, such as noble metals or transition metals such as nickel. The composition of these bimetallic catalysts must be tuned to achieve high activity but also a stability improvement. There are many efforts described in the literature to tune the composition of these catalysts, but other compositions avoiding the use of noble metals should be investigated. In such cases, further investigation of the nature of the bimetallic phases is mandatory to determine the actual phase acting in the catalytic reaction.

The study of the mechanisms must also be deepened. Many studies are focused on the role of the Cu in the reaction; however, the presence of acid or basic sites on the support also plays an important role in the reaction. These studies are not yet reported in the literature due to the fact that the presence of these acid sites modifies the electronic density of the metal but also promotes its interaction with the solvent and FUR without the need for the metal. As CHT reaction leads to the oxidation of the sacrificing alcohol into aldehyde or ketone, these ketones together with FUR can react via acetalization or aldol condensation under the presence of acid or basic sites, causing a blockage of the activity and hindering its regeneration. In addition, the formation of such undesired products implies an increase in cost in the purification stage of the products. Therefore, the election of the support must be performed carefully and with the adequate acid/basic properties.

On the other hand, gas-phase studies are carried out continuously; however, the main drawback is the high deactivation of the catalysts by the formation on the active sites of carbonaceous deposits, so catalysts with higher stability for longer time-on-stream are necessary; therefore, it is necessary to perform studies at long reaction times. Regarding liquid-phase processes, the opposite is true; most of the studies are performed in batch reactors, so the challenge is the design of continuous processes for large-scale production. In order to develop more sustainable processes, the recovery and reuse of the solvent is also important.

Many studies are conducted using commercial purified FUR, but FUR is commercially available as a molecule obtained from lignocellulose raw materials. The FUR obtained in this way contains impurities and the solvent is H_2_O. The study of these real liquors causes a clear impoverishment of the yields due to the presence of undesired products leading to uncontrolled polymerization reactions. Moreover, the H_2_O can oxidize the reduced copper species, leading to a deactivation of the catalyst. Therefore, robust copper catalysts are needed to work in real conditions. In addition, the real FUR dissolutions contain formic or acetic acid, which favor the leaching of the metallic particles, causing the need to neutralize the liquors.

In summary, the development of an efficient Cu-based catalyst able to produce such molecules from FUR should utilize carbohydrates or hemicellulose/cellulose as feedstock, to reduce the formation of byproducts and improve their stability.

## 5. Conclusions

This review summarized the main catalytic processes for the production of relevant chemicals from hydrogenation of furfural (FUR) and levulinic acid (LA), two platform molecules derived from lignocellulosic sugars, focused on the use of copper-based catalysts. Thus, the production of furfuryl alcohol, 2-methylfuran, and cyclopentanone from FUR, and γ-valerolactone and 2-methyltetrahydrofuran from LA, was reviewed. Special emphasis was placed on the most outstanding catalytic results, highlighting the main heterogeneous catalytic processes used (gas- and liquid-phase, catalytic transfer hydrogenation). In addition, the advantages and drawbacks of these catalytic process were identified, and the effect of adding a second metal to prepare bimetallic MCu-based catalysts was considered, as well as the issue related to catalyst stability, considering that a key advantage of heterogeneous catalysts is their reusability. Finally, it is necessary to develop catalytic processes to produce these molecules directly from lignocellulosic biomass, so bifunctional catalysts are required to directly transform carbohydrates in these relevant chemicals in one-pot processes.

## Figures and Tables

**Table 1:** Gas-phase hydrogenation of FUR to FOL and MF using Cu-based catalysts and hydrogen as reductant.

Catalyst	Space Velocity (h^−1^)	H_2_/FUR Molar Ratio	T (°C)	TOS (h)	C ^a^ (%)	Y ^b^ (%)	Ref.
CuCr_2_O_7_	52 (WHSV)	25	200	4	22	20 (FOL)	[51]
Cu/SiO_2_	2.3 (WHSV)	25	290	0.25	77	63 (FOL)	[55]
Cu/SiO_2_	0.5 (WHSV)	17	140	10	90	73 (FOL)	[61]
Cu/SiO_2_	0.5 (WHSV)	17	220	210	100	89.5 (MF)	[61]
Cu/SiO_2_	1.5 (WHSV)	11.5	170	1	91	85 (FOL)	[77]
Cu/SiO_2_	1.5(WHSV)	11.5	210	14	95	80 (MF)	[62]
Cu/Sep ^c^	1.5 (WHSV)	11.5	210	5	83	72 (FOL)	[78]
Cu/Ker ^d^	1.5 (WHSV)	11.5	190	5	91	50 (MF)	[79]
CuCo/SiO_2_	3.1 (WHSV)	6	200	12	65	64 (FOL)	[80]
CuCa/SiO_2_	0.33 (LHSV)	5.1	130	80	100	98 (FOL)	[81]
CuPd/Zeo ^e^	7.7 (WHSV)	0.08	300	-	58	58 (FOL)	[82]
Cu/MgO	4.8 (WHSV)	2.5	180	5	98	96 (FOL)	[65]
Cu/MgO/Sep ^c^	1.5 (WHSV)	11.5	210	5	73	64 (FOL)	[83]
CuCa/MgO	1.7 (WHSV)	10	180	0.5	91	90 (FOL)	[69]
Cu/ZnO	0.5 (WHSV)	17	220	10	95	31 (FOL)	[61]
Cu/ZnO	1.5 (WHSV)	11.5	210	5	93	76 (FOL)	[70]
Cu/ZnO	1.5 (LHSV)	15	200	16	100	94 (MF)	[71]
Cu/ZnO/Al_2_O_3_	0.5 (LHSV)	15	120	16	97	94 (FOL)	[73]
Cu/ZnO/Al_2_O_3_	3.6 (LHSV)	15	200	30	76	73 (FOL)	[74]
Cu/ZnO/Ker ^d^	1.5 (WHSV)	11.5	190	5	62	55 (FOL)	[79]
Cu/ZnO/Sep ^c^	1.5 (WHSV)	11.5	210	5	81	58 (FOL)	[83]
Cu/CeO_2_	1.5 (WHSV)	11.5	190	5	81	67 (FOL)	[75]
Cu/CeO_2_/Al_2_O_3_	60 (W/F)	-	175	6	90	72 (FOL)	[76]
Cu/CeO_2_/Ker ^d^	1.5 (WHSV)	11.5	190	5	64	61 (FOL)	[79]
Cu/CeO_2_/Sep ^c^	1.5 (WHSV)	11.5	210	5	66	61 (FOL)	[83]

^a^ C: conversion; ^b^ Y: yield; ^c^ Sep: sepiolite; ^d^ Ker: kerolite; ^e^ Zeo: zeolite.

**Table 4:** Catalytic activity of Cu-based catalysts for hydrogenation of LA to GVL.

Catalyst	Hydrogen Source	T (°C)	P (MPa)	t (h)	C ^a^ (%)	y_GVL_^b^ (%)	Ref.
CuO–Cr_2_O_3_	H_2_	273	10.1	-	-	62	[170]
Cu–ZrO_2_	H_2_	200	3.4	5	100	100	[11]
Cu–Al_2_O_3_	H_2_	200	3.4	5	100	100	[11]
Cu–ZrO_2_	H_2_	200	3.5	2	100	80	[171]
Cu–ZrO_2_	H_2_	200	3.5	2	100	100	[29]
Mn/Cu–ZrO_2_	H_2_	200	2.6	0.5	82	82	[172]
Ni–Cu/Al_2_O_3_	H_2_	250	6.4	2	100	96	[173]
CuAl_2_O_4_	H_2_	200	6.9	10	98	87	[124]
CuCr_2_O_4_	H_2_	200	6.9	10	>99	91	[124]
CuFe_2_O_4_	H_2_	200	6.9	10	>99	82	[124]
Cu/Al	H_2_	140	3.0	3	76	58	[174]
Cu/Mg/Al	H_2_	140	3.0	3	100	82	[174]
Cu/Ni/Mg/Al	H_2_	140	3.0	3	100	100	[174]
CuAg/Al_2_O_3_	H_2_	180	1.4	4	100	>99	[175]
Cu/CuO–FC	FA ^c^	170	8.4	6	99	50	[176]
CuO–SiO_2_	H_2_	290	0.5	67	>99	93	[112]
Cu/Al_2_O_3_	H_2_	265	0.1	4	98	85	[113]
Cu/ZrO_2_	H_2_	265	0.1	4	81	67	[177]
Cu–ZnO	H_2_	240	1.0	20	-	70	[178]
Cu/SiO_2_	H_2_	250	0.1	5	73	55	[179]
Cu/Al_2_O_3_	H_2_	250	0.1	5	98	95	[179]
Cu–Ni/SiO_2_	H_2_	250	0.1	-	98	96	[20]
Cu/SiO_2_	H_2_	265	1.0	100	100	99.9	[14]
Ni–Cu/SiO_2_	FA ^c^	265	0.1	100	98	90	[180]
Cu–Ni/SiO_2_	H_2_	250	0.1	50	100	99	[181]
Cu/SiO_2_	FA ^c^	270	0.1	-	66	53	[182]
Cu/SiO_2_	FA ^c^	250	0.1	-	56	49	[183]
Cu/Fe_2_O_3_	FA ^c^	250	0.1	3	100	100	[184]

^a^ C: conversion; ^b^ y_GVL_: GVL yield; ^c^ FA: formic acid.

**Table 5:** Catalytic activity of Cu-based catalysts for hydrogenation of LA to GVL in the presence of alcohols.

Catalyst	Solvent	Hydrogen Source	T (°C)	P (MPa)	T (h)	C ^a^ (%)	y_GVL_^b^ (%)	Ref.
Cu–ZrO_2_	Methanol	H_2_	200	3.4	5	100	90	[11]
Magnetic Ni/Cu/Mg/Al/Fe	Methanol	H_2_	142	2	3	100	98	[190]
Cu/Al_2_O_3_	Methanol	H_2_	24	0.1	5	94	86	[179]
Cu/γ-Al_2_O_3_	Ethanol	H_2_	200	4.9	6	100	93	[23]
Cu–WO_3_/ZrO_2_	Ethanol	H_2_	200	4.9	6	100	94	[23]
Cu	Methanol	Methanol	240	—	1	97	85	[21]
CuCr_2_O_4_	Methanol	Methanol	250	0.1	4	94	96	[191]
Cu–MgO	Methanol	Methanol	220	—	4	96	91	[192]
CuO_x_–CaCO_3_	Methanol	Methanol	240	—	3	>99	96	[193]
Cu/C	2-Propanol	2-Propanol	220	2.0	5	>99	89	[10]

^a^ C: conversion; ^b^ y_GVL_: GVL yield.

## Data Availability

Not applicable.
